# Comprehensive effects of plastic film mulching and combined application of water and nitrogen on photosynthetic characteristics, yield formation, and water-nitrogen use efficiency of citrus

**DOI:** 10.3389/fpls.2026.1963480

**Published:** 2026-10-12

**Authors:** Hongmei Gu, Ping Li, Mengyuan Deng, Yun Zhong, Yuliang Fu, Songlin Wang

**Affiliations:** 1 School of Water Conservancy, North China University of Water Resources and Electric Power, Zhengzhou, China; 2 Soil and Water Conservation Supervision Bureau in the contiguous areas of Shanxi, Shaanxi and Inner Mongolia, Yulin, China; 3 College of Hydraulic and Environmental Engineering, China Three Gorges University, Yichang, China

**Keywords:** citrus, PLS-PM, response surface optimization, surface mulching, water-nitrogen coupling

## Abstract

**Introduction:**

Citrus production in western Hubei, a subtropical region of central China, is constrained by seasonal water scarcity and excessive nitrogen application, creating a major challenge in reducing water and nitrogen inputs while maintaining yield stability and improving resource-use efficiency. Moreover, how different mulching materials modulate citrus responses under deficit irrigation and reduced nitrogen application remains unclear. Therefore, a multifactor field experiment integrating mulching type, irrigation, and nitrogen application was conducted to evaluate their combined effects on citrus photosynthetic traits, yield formation, water-use efficiency (WUE), and nitrogen partial factor productivity (NPFP).

**Methods:**

The field experiment was conducted in Zhijiang, Hubei Province, China, in 2023 and 2024 using an L9(3^4^) orthogonal design, incorporating three irrigation levels (W1: 80% *θf* - 90% *θf*; W2: 70% *θf* - 80% *θf*; W3: 60% *θf* - 70% *θf*, where *θf* denotes field capacity, defined as the soil water content retained after gravitational water has drained from fully wetted soil), three nitrogen rates (N1: 80% N0; N2: 70% N0; N3: 60% N0, where N0 = 300 kg·ha^-1^), and three mulching materials (M1: white transparent film; M2: black opaque film; M3: silver-black double-color reflective film).

**Results:**

(1) Irrigation amount (W) and nitrogen rate (N) were the primary factors influencing the measured variables, whereas mulching material (M) had a comparatively smaller effect. The main effects of all three factors were significant for most variables (*P* < 0.05). Interaction analyses indicated significant W × M and N × M effects on most variables, whereas W × N effects were comparatively limited. (2) As water and nitrogen supply decreased, Pn, Tr, Gs, and CE declined concurrently. Compared with T1 (W1N1M1), Pn decreased by 1.02%–24.36% under the other treatments. Ci declined in parallel with Pn under W1 and W2 but increased under W3. Fruit number and single-fruit weight responded differently to irrigation, nitrogen application, and mulching, The two variables reached their highest values under T1 and T5, respectively, and were significantly higher than their corresponding values under all W3 treatments (*P* < 0.05). (3) Using the criterion that yield, WUE, and NPFP each reached at least 90% of its respective maximum, the model-estimated suitable water and nitrogen ranges within the experimental domain were M1 (W = 687–800 mm, N = 180–208 kg·ha^-1^), M2 (W = 706–740 mm, N = 219–237 kg·ha^-1^), and M3 (W = 776–800 mm, N = 205–226 kg·ha^-1^). (4) Path analysis and PLS-PM yielded positive path coefficients from irrigation, nitrogen application, and mulching material to photosynthetic traits (*β* = 0.87, 0.33, and 0.05, respectively), from photosynthetic traits to growth indicators (*β* = 0.93), and from growth indicators to yield (*β* = 0.78). Fruit number had a greater path coefficient for yield than single-fruit weight.

**Discussion:**

These findings provide a theoretical basis and quantitative practical references for the selection of mulching materials and precise water-nitrogen management in citrus under deficit irrigation and reduced nitrogen fertilization in Western Hubei.

## 1 Introduction

Citrus is one of the most extensively cultivated and productive fruit crops worldwide. According to FAOSTAT, global citrus production reached approximately 165.5 million tonnes in 2024, playing an important role in regional agricultural economies and farmers’ income generation (FAO, 2025). In western Hubei, China, citrus production is constrained by both seasonal water scarcity and excessive nitrogen application, creating a pronounced trade-off between reducing water and nitrogen inputs and maintaining stable yields and improving resource-use efficiency. Seasonal drought in this region occurs primarily during the summer fruit-expansion stage, causing tree water deficits and restricting fruit development (Xian et al., 2022). Meanwhile, excessive nitrogen application in pursuit of high yields reduces nitrogen -use efficiency, wastes resources, and aggravates environmental problems such as soil acidification and water eutrophication (Luo et al., 2023; Zhu et al., 2022). Studies have shown that nitrogen losses through leaching, runoff, and gaseous emissions account for 24%–35% of total nitrogen inputs in citrus orchards (Luo et al., 2023). Therefore, the key challenge for citrus production in western Hubei is not simply to reduce irrigation or nitrogen application, but to clarify crop responses to water and nitrogen and optimize their spatiotemporal allocation, thereby maintaining stable yields and improving resource-use efficiency with lower water and nitrogen inputs.

The essence of water-nitrogen coupling lies in dynamically regulating the timing and proportions of water and fertilizer supply according to the differentiated demands of citrus at different growth stages, ensuring precise alignment of resource allocation with tree physiological requirements, thereby improving water-use efficiency (WUE) and nitrogen partial factor productivity (NPFP) under resource-limited conditions. Studies have shown that moderate deficit irrigation and reduced N application can significantly enhance WUE and NPFP while maintaining the potential for high yields. However, excessive reductions in water and nitrogen may lead to irreversible yield losses, a problem that is particularly pronounced in arid regions (Zhang et al., 2026; Fan et al., 2026). Precise coordination of water and nitrogen in citrus aims to drive simultaneous improvements in photosynthetic capacity and root uptake by matching water and nitrogen in terms of quantity and timing. When the two are appropriately matched, leaves can synthesize sufficient photosynthetic products and roots can efficiently absorb nutrients, jointly promoting carbon assimilation and nutrient accumulation in the tree, ultimately manifesting as vigorous shoot growth, enlarged fruit, and increased yield. However, findings regarding water-nitrogen coupling remain inconsistent and can be broadly classified into two categories based on photosynthetic responses. One group of studies showed that an appropriate combination of irrigation and nitrogen supply maintained or increased net photosynthetic rate (Pn) despite reduced stomatal conductance (Gs) (He et al., 2025; Li et al., 2025). In contrast, another group reported that water deficit combined with reduced nitrogen rate simultaneously decreased Pn, transpiration rate (Tr), and Gs, while increasing only leaf instantaneous water-use efficiency (LWUE) (Chen et al., 2026). Additionally, water and nitrogen supply are particularly crucial for New shoot elongation and fruit diameter expansion. Appropriate water-nitrogen ratios can promote vigorous shoot growth and expand leaf area, providing ample assimilates (“sources”) for fruit enlargement (“sinks”); however, excessive water and nitrogen can easily induce excessive vegetative growth, causing the excessive consumption of assimilates by vegetative organs and reducing the proportion allocated to fruits, thereby inhibiting fruit enlargement and dry matter accumulation. Conversely, insufficient water and nitrogen restrict shoot development, reduce leaf area, decrease total photosynthetic carbon assimilation, and ultimately limit fruit development due to carbon source deficiency (Kumar et al., 2025).

The introduction of plastic film mulching provides an additional micro-environmental regulatory variable for water-nitrogen management. White transparent film can enhance light use efficiency by increasing photosynthetically active radiation in the lower canopy; black opaque film can reduce non-productive water loss by suppressing soil surface evaporation; and silver-black double-color reflective film is designed to integrate both light-enhancing and moisture-conserving functions. Previous studies have shown that plastic film mulching can significantly increase yield and WUE, with the magnitude of enhancement varying among mulching types, and that significant interactive effects on yield and WUE exist between deficit irrigation and mulching (Zhong et al., 2025). Furthermore, black film mulching combined with full irrigation can effectively increase plant height, expand canopy width, and thereby improve photosynthetic rate (Choudhary et al., 2024). Plastic film mulching can also alter nitrogen transformation and migration in soil, with some studies reporting that mulching increases nitrogen partial factor productivity by 26%, although the responses to “mulching-nitrogen coupling” differ significantly among crop species (Huang et al., 2024). However, existing studies on citrus cultivation have largely focused on pairwise interactions among mulching, irrigation, and nitrogen fertilization, whereas integrated studies incorporating all three factors remain scarce. More critically, the simultaneous optimization of multi-objective water-nitrogen intervals that balance yield, WUE, and NPFP has not been validated under field conditions. Furthermore, the complete pathway through which different mulching materials drive vegetative growth via photosynthetic physiology and ultimately influence yield formation under deficit irrigation and reduced nitrogen fertilization has yet to be quantitatively elucidated.

Based on previous studies, we proposed the following hypotheses: (H1) moderate deficit irrigation combined with reduced nitrogen fertilization can improve WUE and NPFP while maintaining relatively high yields; (H2) mulching materials differ in their capacity to buffer the effects of reduced water and nitrogen inputs, with silver–black reflective mulch being more effective in maintaining citrus photosynthetic capacity, growth, and yield; and (H3) irrigation, nitrogen application, and mulching indirectly affect yield formation primarily by regulating photosynthetic traits and plant growth. To test these hypotheses, this study employed an L9(3^4^) orthogonal experimental design with three deficit irrigation levels, three reduced nitrogen levels, and three mulching materials to systematically analyze the regulatory effects of water-nitrogen-mulching coupling on citrus physiological growth, yield formation, and water-nitrogen use efficiency. The specific objectives were: (1) to elucidate the independent and interactive effects of deficit irrigation, reduced nitrogen application, and mulching materials on citrus leaf photosynthetic pigments, antioxidant enzyme activities, photosynthetic parameters, and new shoot and fruit diameter growth; (2) to determine the comprehensive water-nitrogen management intervals that ensure both high yield and high efficiency under different mulching conditions based on a second-order polynomial regression model combined with spatial overlay analysis; (3) to analyze the internal causal structure of yield formation and the complete pathway through which treatment factors drive yield via physiological mediators using observed-variable path analysis and Partial Least Squares Path Modeling (PLS-PM), and to quantify the relative contributions of fruit number and single fruit weight to yield. This study aims to integrate in-depth mechanistic analysis of citrus physiology with field water-nitrogen-mulching management practices, providing a theoretical foundation and quantitative practical reference for the precise regulation of deficit irrigation and reduced nitrogen fertilization in citrus in seasonally arid regions.

## 2 Materials and methods

### 2.1 Description of the experimental site

The experiment was conducted from March to November in 2023 and 2024 at the Citrus Irrigation Experimental Base of China Three Gorges University (Sigang Village), located in Zhijiang City, Yichang City, Hubei Province, China (111°48′E, 30°36′N; altitude 46 m) (**Figure 1**). The experimental area has a subtropical monsoon climate with a frost-free period of 270 days. The soil type is sandy loam developed from Quaternary red clay, exhibiting typical ecological characteristics of the Western Hubei citrus-producing region. The experiment covered the entire annual growth cycle of citrus (from budding and flowering to fruit maturity). The base is equipped with an automatic weather station (monitoring wind speed, temperature, humidity, and photosynthetically active radiation) and a soil moisture monitoring system, providing precise control conditions for the water-nitrogen combined application experiment. The basic physicochemical properties of the sandy loam soil are shown in **Table 1**. The soil is acidic, with a pH range of 4.8-6.2, bulk density of 1.38 g·cm^-3^, and field capacity of 20% (mass water content). The organic matter content was 19.09 g·kg^-1^. Total nitrogen, total phosphorus, total potassium, hydrolyzable nitrogen, available phosphorus, and available potassium were 1.02 g·kg^-1^, 0.53 g·kg^-1^, 16.18 g·kg^-1^, 125.98 mg·kg^-1^, 21.33 mg·kg^-1^, and 144.46 mg·kg^-1^, respectively. From March 1 to December 1, 2023, the total rainfall was 1887.80 mm, with daily maximum and minimum temperatures of 37.7 °C and 3.3 °C, respectively. During the same period in 2024, the rainfall was 1412.4 mm, with maximum and minimum temperatures of 40.1 °C and 2.6 °C, respectively. The climate is a subtropical continental monsoon climate, characterized by hot, rainy summers and mild, dry winters. Rainfall and air temperature during the experimental period are shown in **Figure 2**.

**Figure 1:**
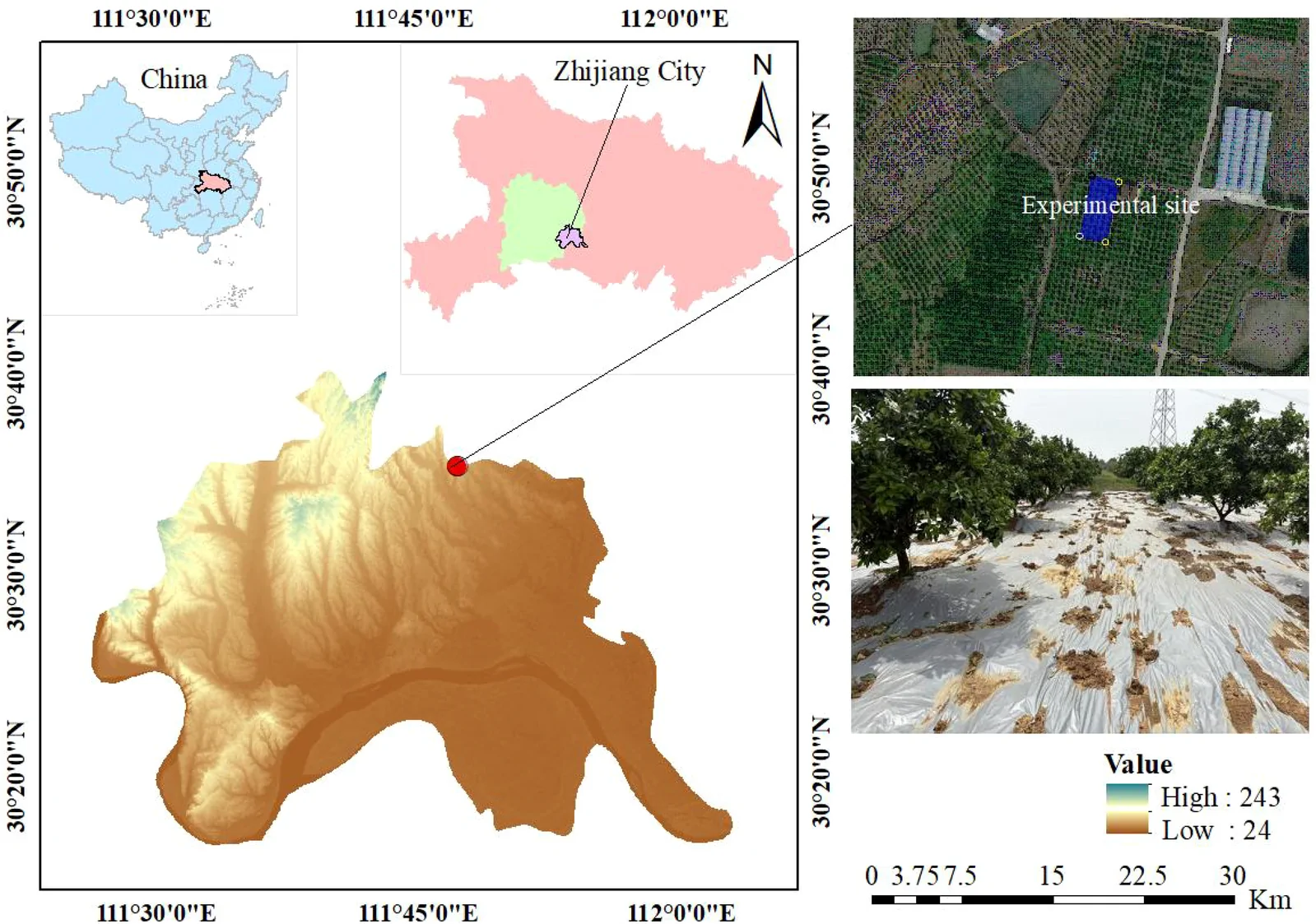
Location of the experimental site.

**Table 1:** Basic physicochemical properties of the soil at the experimental site.

pH range	Bulk density (g·cm^-3^)	Organic matter (g·kg^-1^)	Total nitrogen (g·kg^-1^)	Total phosphorus (g·kg^-1^)	Total potassium (g·kg^-1^)	Hydrolyzable nitrogen (mg·kg^-1^)	Available phosphorus (mg·kg^-1^)	Available potassium (mg·kg^-1^)
4.8-6.2	1.38	19.09	1.02	0.53	16.18	125.98	21.33	144.46

**Figure 2:**
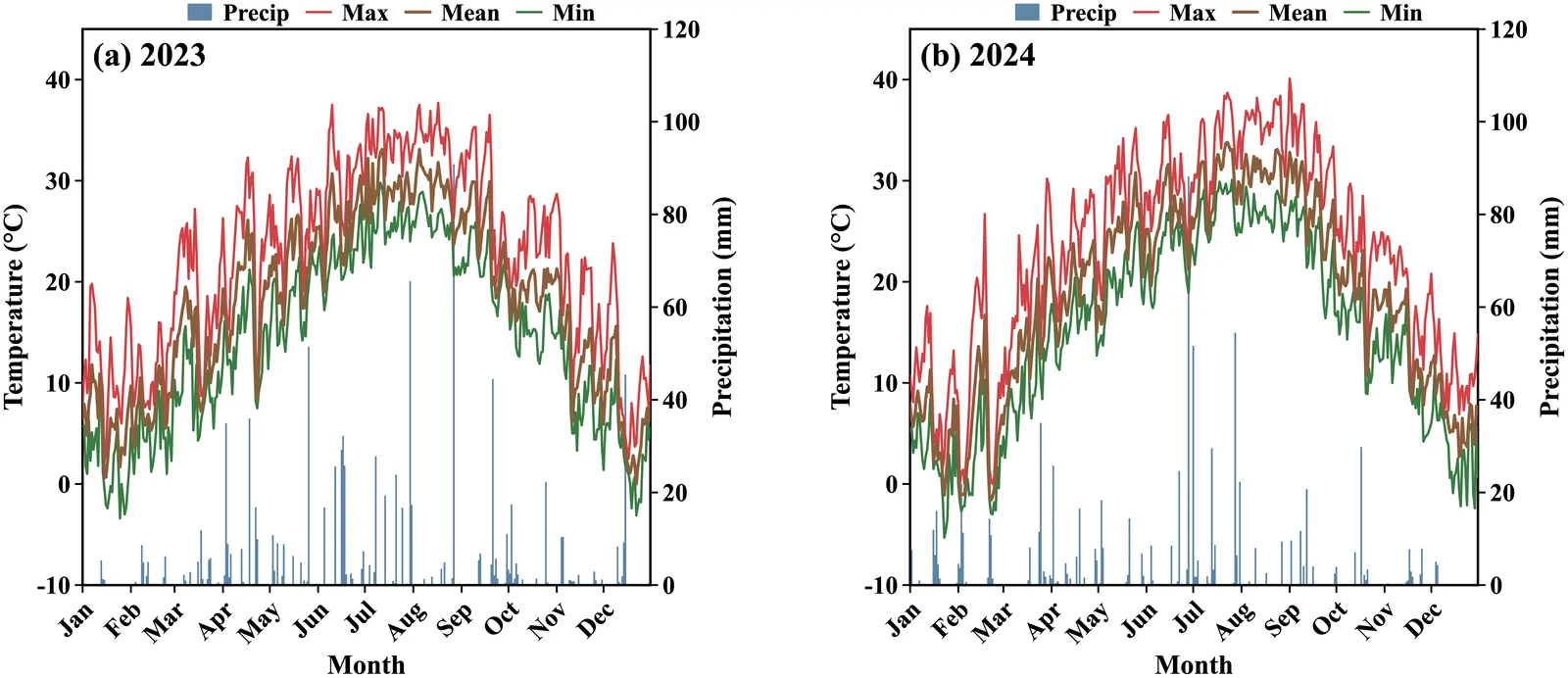
Daily air temperature and precipitation at the experimental site from March to November during 2023-2024. Panels **(a, b)** represent air temperature and precipitation in 2023 and 2024, respectively.

### 2.2 Experimental design

Six-year-old ‘Newhall’ navel orange trees grafted onto trifoliate orange (*Poncirus trifoliata* [L.] Raf.) rootstocks were used as the experimental material. The trees were 167–219 cm tall, with trunk diameters of 5.6–9.6 cm. The planting rows were oriented north–south, and the average canopy width ranged from 190 to 230 cm. The trees were planted at a spacing of 4 m × 2 m, corresponding to a planting density of 1,250 trees ha^-1^. At the beginning of the experiment, the trees assigned to the different treatments had comparable vigor and canopy architecture. Before the experiment, the orchard was managed uniformly following conventional local practices, without mulching or treatment-specific irrigation and nitrogen management. Based on field observations in 2023–2024, the phenological stages of ‘Newhall’ navel orange were divided into budding and flowering stage (Stage I, mid-March to late April), young fruit stage (Stage II, early May to late May), fruit enlargement stage (Stage III, early June to mid-September), and fruit coloration and sugar-accumulating stage (Stage IV, late September to late October). Preliminary research determined that ‘Newhall’ navel orange could survive when soil moisture was between 60% *θf* and 90% *θf*. Therefore, this experiment set three water stress levels: mild (W1), moderate (W2), and severe (W3), corresponding to soil moisture contents of 80% *θf* - 90% *θf*, 70% *θf* - 80% *θf*, and 60% *θf* - 70% *θf*, respectively. Irrigation was initiated when the mean volumetric soil water content within the 0–60 cm soil layer approached or fell below the lower threshold of the corresponding water treatment and was continued until the upper threshold was reached. Drip irrigation under mulch was used, with irrigation amount and flow rate controlled by flow meters and pressure gauges. Drippers were placed 0.4 m from the tree base in four directions, with a flow rate of 2 L·h^-1^ and operating pressure of 0.1 MPa.

The mulching materials were selected based on their distinct functional properties. The white transparent film can improve canopy light-use efficiency by increasing photosynthetically active radiation in the lower canopy; the black opaque film can effectively suppress soil-surface evaporation and reduce nonproductive water loss; and the silver–black reflective film was designed to combine enhanced light availability with soil-moisture conservation. Accordingly, three mulching materials commonly used in the local area were included as the mulch treatments: white transparent film (M1), black opaque film (M2), and silver–black reflective film (M3), each measuring 100 m × 2 m × 0.02 mm (length × width × thickness). The films were installed during the budding and flowering stage each year and remained in place until fruit harvest was completed. They were then removed and collected, and any residual film was promptly cleared from the experimental area to ensure that no visible plastic residues remained on the soil surface. New films corresponding to the original treatments were installed in the following year. The film-installation procedure was identical for all treatments. Before installation, the orchard floor was levelled, and only shallow tillage (≤8 cm) was conducted between the tree rows. Large stones and weeds were removed, and drainage ditches were constructed around the mulched area. The films were stretched taut, laid flat in close contact with the soil surface, and secured along the edges using stones and soil-filled bags. Rainwater was directed from the film surface into the drainage ditches.

Based on pre-trial surveys, the local fertilization regime for ‘Newhall’ navel orange involved three applications per year. The fixed annual application rates were P_2_O_5_ (206 kg·ha^-1^), K_2_O (337 kg·ha^-1^), and urea (300 kg·ha^-1^). Urea (N 46%), superphosphate (P_2_O_5_ 12%), and potassium sulfate (K_2_O 52%) were used as N, P, and K fertilizers, respectively. Before the budding and flowering stage, all P_2_O_5_, 50% of N, and 33.4% of K_2_O were applied as basal fertilizer. During the budding and flowering stage and the young fruit stage, 15% of N was applied each time. During the fruit enlargement stage, 20% of N and 66.6% of K_2_O were applied. Based on the actual local practice of excessive fertilization, the nitrogen application range was set at 60%-80% of the conventional local rate. The 10% reduction gradient was chosen to clearly distinguish treatment effects and avoid data overlap from densely packed gradients. Thus, three nitrogen reduction levels were established relative to the conventional urea rate: N1: 20% reduction (240 kg·ha^-1^), N2: 30% reduction (210 kg·ha^-1^), and N3: 40% reduction (180 kg·ha^-1^). This 20%-40% reduction gradient allowed quantification of the effects of different nitrogen reduction levels on citrus yield, aiming to break the local redundancy in nitrogen fertilization and achieve fertilizer reduction with increased efficiency. Fertilizer was applied in ring-shaped trenches dug along the drip line of the experimental trees.

All treatments followed the same standard agronomic practices, except for water, fertilizer, and mulching materials, including pruning, girdling, pesticide spraying, and weed control. Given that orthogonal design efficiently evaluates the main effects of irrigation, nitrogen, and mulching while reducing experimental workload, the experiment utilized an L9(3^4^) orthogonal array design with three factors: irrigation amount (W), nitrogen rate (N), and mulching material (M), resulting in nine treatments (T1–T9). Each treatment was replicated three times, resulting in 27 experimental plots. Each plot contained three citrus trees, giving a total of 81 trees. The experimental scheme is shown in **Table 2**.

**Table 2:** Experimental design for the combined application of water and nitrogen under mulching treatments.

Treatments	Deficit irrigation (W)	Nitrogen fertilizer reduction(N)	Mulching film material (M)	Level combination
T1	1(80% *θf* - 90% *θf*)	1(240 kg·ha^-1^)	1(white transparent film)	W1N1M1
T2	1(80% *θf* - 90% *θf*)	2(210 kg·ha^-1^)	2(black opaque film)	W1N2M2
T3	1(80% *θf* - 90% *θf*)	3(180 kg·ha^-1^)	3(silver-black double-color reflective film)	W1N3M3
T4	2(70% *θf* - 80% *θf*)	1(240 kg·ha^-1^)	2(black opaque film)	W2N1M2
T5	2(70% *θf* - 80% *θf*)	2(210 kg·ha^-1^)	3(silver-black double-color reflective film)	W2N2M3
T6	2(70% *θf* - 80% *θf*)	3(180 kg·ha^-1^)	1(white transparent film)	W2N3M1
T7	3(60% *θf* - 70% *θf*)	1(240 kg·ha^-1^)	3(silver-black double-color reflective film)	W3N1M3
T8	3(60% *θf* - 70% *θf*)	2(210 kg·ha^-1^)	1(white transparent film)	W3N2M1
T9	3(60% *θf* - 70% *θf*)	3(180 kg·ha^-1^)	2(black opaque film)	W3N3M2

### 2.3 Measurements and methods

#### 2.3.1 Soil moisture measurement

Field capacity (*θf*) was determined using the core method. Before the experiment, undisturbed soil cores were collected separately from the 0–20, 20–40, and 40–60 cm soil layers using 100 cm³ core samplers. The samples were saturated with distilled water for 24 h and then placed on dry sand to drain for 48 h until gravitational water had largely drained. Subsequently, the samples were oven-dried at 105 °C for 24 h to determine gravimetric water content, which was converted to volumetric water content using soil bulk density (**Table 1**); the resulting value was taken as field capacity. During the budding-flowering, young-fruit, fruit-expansion, and fruit-coloring and sugar-accumulation stages, soil samples were collected by layer using a soil auger. Gravimetric water content was determined by the same oven-drying method and converted to volumetric water content for irrigation-amount calculations and monitoring soil-water dynamics.

#### 2.3.2 Citrus physiological and growth measurements

During the experiment, new shoot length, fruit diameter, and leaf photosynthetic parameters were measured on the selected trees. The mean of the three trees within each plot was used as the plot-level observation. Each plot was considered an experimental unit, with three independent replicate plots per treatment (*n* = 3) for statistical analysis.

##### 2.3.2.1 Chlorophyll and antioxidant enzymes

To minimize transient physiological fluctuations caused by the rapid redistribution of soil water after irrigation and to compare physiological responses among treatments under relatively stable soil moisture conditions, leaves were sampled 72 h after each irrigation event. From each of the three permanently designated trees in each plot, 8–10 healthy, pest- and disease-free mature leaves were collected from the middle portions of shoots distributed across the eastern, southern, western, and northern sides of the canopy. Leaves of comparable age were selected, whereas newly emerged and senescent leaves were excluded. All leaves collected from the same plot were pooled to form one composite sample and immediately snap-frozen in liquid nitrogen to rapidly suppress tissue metabolic activity. After transport to the laboratory, the samples were stored in darkness at -80 °C to minimize chlorophyll degradation and changes in superoxide dismutase (SOD), catalase (CAT), and peroxidase (POD) activities. All measurements were completed within one week. Chlorophyll content was determined using the 96% ethanol extraction method and quantified spectrophotometrically. SOD activity was measured using the nitroblue tetrazolium (NBT) photoreduction method, with absorbance recorded at 560 nm. One unit of SOD activity was defined as the amount of enzyme required to inhibit NBT photoreduction by 50%, and the results were expressed as U·g^-1^ FW. CAT activity was determined using the ultraviolet absorption method, with absorbance recorded at 240 nm. POD activity was determined using the guaiacol method, with absorbance recorded at 470 nm. Both CAT and POD activities were expressed as U·g^-1^ FW·min^-1^. Detailed measurement and calculation procedures are provided in Reference (Darvizheh et al., 2019).

##### 2.3.2.2 New shoot length

Owing to interannual variation in meteorological conditions, the timing of new shoot emergence differed between years. To ensure that observations began at the same phenological stage in both years, shoot labeling and measurements commenced on 6 July 2023 and 3 July 2024, corresponding to the initial stage of new shoot emergence in each year. On each permanently designated tree, 3–5 vigorously growing new shoots with similar orientations were selected across the eastern, southern, western, and northern sides of the canopy and labeled with numbered red plastic tags. A total of 9–15 shoots were labeled in each plot. Beginning on the labeling date, the absolute length of each marked shoot from the base to the tip was measured every 5 d using a steel measuring tape with a precision of 1 mm, and the measurements were used to calculate shoot growth rates. Observations were conducted from 6 July to 4 October in 2023 and from 3 July to 1 October in 2024, with each observation period lasting 90 d. At each measurement, the arithmetic mean length of all marked shoots on a tree was used as the value for that tree, and the mean of the three trees was used as the plot-level value.

##### 2.3.2.3 Fruit diameter

On 30 April 2023 and 2 May 2024, at the early stage of young fruit development following full bloom, when the young fruits were primarily undergoing cell division and physiological fruit drop, 3–5 representative fruits of uniform size, regular shape, and free of pests and diseases were selected from the eastern, southern, western, and northern directions of the canopy of each marked tree. The longitudinal and transverse diameters were measured every 5 days using a vernier caliper with an accuracy of 1 mm, measurements continued until harvest. At each measurement date, the longitudinal and transverse diameters of all tagged fruits on each tree were averaged separately to obtain the corresponding values for that tree. The respective means of the three observation trees within each plot were then calculated as the final plot-level values. Longitudinal and transverse fruit diameters were analyzed and reported separately.

##### 2.3.2.4 Photosynthetic parameters

On clear days following irrigation, leaf gas-exchange characteristics were measured at 2-h intervals from 09:00 to 17:00 under natural light using a portable photosynthesis system. Net photosynthetic rate (*Pn*), transpiration rate (*Tr*), stomatal conductance (*Gs*), and intercellular CO_2_ concentration (*Ci*) were recorded. For each measurement, healthy, fully expanded leaves of comparable maturity and insertion position were selected from the middle portion of the same shoot flush, while newly emerged and senescent leaves were excluded. Three representative leaves were measured on each designated tree, and three consecutive stable readings were recorded for each leaf. All measurements obtained from the three observation trees within each plot were averaged to generate a representative plot-level value for subsequent statistical analyses.

##### 2.3.2.5 Leaf Instantaneous water use efficiency and carboxylation efficiency

Leaf instantaneous water use efficiency (*LWUE*, μmol·mmol^-1^), defined as the ratio of net photosynthetic rate to transpiration rate, was calculated as follows (Equation 1):

(1) LWUE=Pn/Tr

Carboxylation efficiency (*CE*, mol·m^-2^·s^-1^), defined as the ratio of net photosynthetic rate to intercellular CO_2_ concentration, was calculated as follows (Equation 2):

(2) CE=Pn/Ci

where *Pn*, *Tr*, and *Ci* were obtained from simultaneous measurements on the same set of leaves.

##### 2.3.2.6 Water consumption

Irrigation amount (*I*) was calculated as follows (Equation 3):

(3) $$I=0.1\gamma \cdot z\cdot p\cdot S(\theta_{max}-\theta_{min})/\eta$$

Where *I* is the irrigation amount (L)*;* γ is the soil bulk density (1.38 g·cm^-1^);  z is the planned wetting layer depth (0.6 m); *p* is the wetting ratio (0.3); *S* is the area per tree (m^2^, determined by measurement); $\;\theta_{max}$ and $\theta_{min}$ are the upper and lower limits of soil moisture (mass water percentage); and η is the irrigation water use efficiency (0.90 for drip irrigation).

Citrus evapotranspiration (*ET*) was calculated using the water balance method (Equation 4):

(4) $$ET=\text{Δ}W+I+P_{r}+G-D-R$$

Where Δ*W* is the reduction in soil water storage between two measurements (mm); *I* is the irrigation amount (mm); *P_r_* is the effective rainfall (mm); Throughout the experimental period, the soil surface remained fully covered with plastic film. Following canopy interception, most rainfall was discharged over the film surface, although a small amount might have infiltrated along the tree trunks. Consequently, rainfall recharge to the designated wetted soil layer was substantially restricted. Because this small amount of infiltration could not be quantified separately, effective rainfall (*P_r_*) was assumed to be zero in the water-balance calculations. This assumption may have resulted in a slight underestimation of crop water consumption; however, all treatments used the same mulching and drainage procedures, thereby limiting its influence on comparisons among treatments treatments; *G* is the groundwater recharge (mm, ignored as groundwater was below 10 m); *D* is deep drainage (mm, ignored for surface drip irrigation with low flow rate and quota); and *R* is surface runoff (mm, ignored due to minimal runoff). Therefore, (Equation 4) was simplified to (Equation 5):

(5) ET=ΔW+I

##### 2.3.2.7 Yield and water-nitrogen use efficiency

Yield (*Y*) was calculated based on the average single fruit weight and average plot area (Alemayehu et al., 2023). Water use efficiency (*WUE*, kg·m^-3^) and nitrogen partial factor productivity (*NPFP*, kg·kg^-1^) were defined as the ratios of yield to water consumption and fertilizer rate, respectively (Fernández, 2023) (Equations 6, 7).

(6) WUE=0.1Y/ET

(7) NPFP=Y/N

### 2.4 Statistical analysis

Data were organized using Microsoft Excel 2019 and statistically analyzed using SPSS 27.0. Figures were prepared using Origin 2024, Python 3.12, and Microsoft Visio. Differences among treatments were assessed using Duncan’s multiple range test (*P* < 0.05). Range analysis was used to evaluate the relative effects of the experimental factors, and multifactor analysis of variance was performed to test their main effects. The W × N, W × M, and N × M interactions were analyzed separately on an exploratory basis.

Path analysis was used to examine the relationships among shoot length, fruit diameter, fruit number, mean fruit weight, and yield (Parmar et al., 2025). After variable standardization, path coefficients (*β*) were estimated equation by equation using the ordinary least-squares method. Direct, indirect, and total effects were decomposed using path-tracing procedures, and the significance of the path coefficients and indirect effects was evaluated using bootstrap resampling with 5,000 iterations. Partial Least Squares Partial least-squares path modeling (PLS-PM) was used to characterize the pathways through which irrigation amount, nitrogen application, and mulching affected yield formation via physiological and growth-related mediators (Su et al., 2026). Irrigation, nitrogen rates, and mulching material were specified as exogenous variables; photosynthetic traits (Pn and Gs) and growth indicators (FSL, FN, and SFW) were specified as mediating constructs; and yield, WUE, and NPFP were specified as outcome variables. The reliability and convergent and discriminant validity of the measurement model were evaluated using outer loadings (>0.80), composite reliability (>0.70), average variance extracted (>0.50), and the heterotrait–monotrait ratio of correlations (HTMT < 0.90). Collinearity in the structural model was assessed using the variance inflation factor (VIF < 5.0), explanatory power was evaluated using *R*², and the significance of the path coefficients was tested using bootstrap resampling. Model fit was assessed using the standardized root mean square residual (SRMR<0.80). The path analysis and PLS-PM were conducted in Python 3.12. Given the limited sample size, the PLS-PM results were interpreted as exploratory, and the identified pathways require further validation through larger-scale experiments.

## 3 Results

### 3.1 Effects of surface plastic film mulching and combined water and nitrogen application on citrus physiological characteristics

#### 3.1.1 Effects on citrus leaf photosynthetic pigments

**Figure 3** illustrates the changes in citrus leaf photosynthetic pigment contents under different mulching and water-nitrogen treatments during 2023-2024. Analysis of variance showed that irrigation amount and nitrogen rate significantly affected chlorophyll a and chlorophyll b in both 2023 and 2024 (*P* < 0.001), whereas the effect of mulching material was significant only in 2023 (*P* < 0.05). Irrigation amount and mulching material significantly affected carotenoid content in both years (*P* < 0.001), while nitrogen rate had no significant effect. All two-way interactions among the three factors significantly affected the three photosynthetic pigments (*P* < 0.05 or *P* < 0.001) **Table 3**. Range analysis indicated that irrigation amount and nitrogen rate had pronounced dominant effects on all photosynthetic pigments, while the influence of mulching materials was relatively minor, with the order of factor importance varying by pigment type. Nitrogen rate was the primary factor affecting chlorophyll a and b (N > W > M), whereas irrigation amount had a dominant effect on carotenoid content (W > M > N). Mulching had a relatively weak impact on photosynthetic pigments. Overall, chlorophyll a and b contents showed decreasing trends as nitrogen levels decreased, with high-nitrogen treatments having substantially higher chlorophyll contents than low-nitrogen treatments; the reduction in irrigation further exacerbated these declines. The chlorophyll a and chlorophyll b contents were highest under T1 (W1N1M1) and were significantly higher than under all other treatments (*P* < 0.05), whereas the lowest values were observed under T9 (W3N3M2). In 2023, chlorophyll a and b contents in the remaining treatments decreased by 14.35%–43.48% and 13.39%–44.88%, respectively, compared with T1, with similar trends in 2024. Carotenoid showed an opposite trend; their contents in the low-irrigation treatments (T7-T9) were higher than in the other treatments(*P* < 0.05), reaching the maximum under severe deficit.

**Figure 3:**
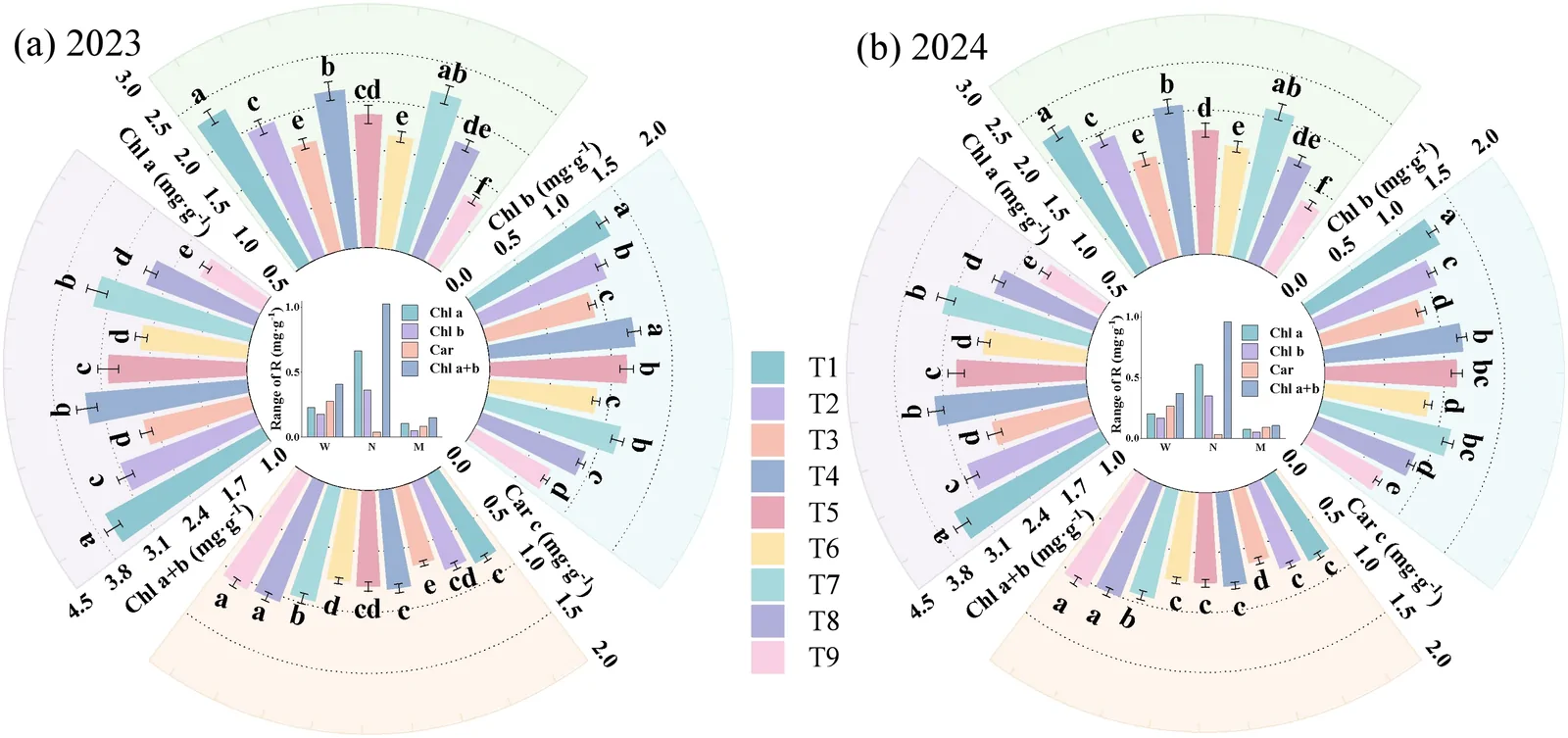
Effects of surface mulching and combined water-nitrogen application on citrus chlorophyll content in 2023-2024. The inner bar chart represents the range (*R*) analysis values, and the outer bar chart represents the mean values of each treatment for different indicators. Different lowercase letters indicate significant differences among treatments (*P* < 0.05). Panels **(a, b)** represent cirus chlorophyll content in 2023 and 2024, respectively.

**Table 3:** ANOVA results for photosynthetic pigment contents in citrus leaves.

Indicators	2023						2024					
	*F*-value			*P*-value			*F*-value			*P*-value		
	W	N	M	W	N	M	W	N	M	W	N	M
Chl a	21.985	188.497	4.975	*<*0.001	*<*0.001	0.018	12.527	108.758	1.747	<0.001	<0.001	0.200
Chl b	108.396	390.989	9.334	*<*0.001	*<*0.001	0.001	42.490	151.754	3.338	<0.001	<0.001	0.056
Car	143.289	3.252	13.248	*<*0.001	0.060	<0.001	104.805	1.482	11.827	<0.001	0.251	<0.001
Chl a + b	45.293	277.000	6.279	*<*0.001	*<*0.001	0.008	21.638	131.027	1.978	<0.001	<0.001	0.164
	W×N	W×M	N×M	W×N	W×M	N×M	W×N	W×M	N×M	W×N	W×M	N×M
Chl a	14.812	251.487	36.749	<0.001	<0.001	<0.001	5.203	93.599	14.108	0.006	<0.001	<0.001
Chl b	10.795	312.718	89.162	<0.001	<0.001	<0.001	4.072	99.450	29.232	0.016	<0.001	<0.001
Car	39.0532	20.326	282.670	<0.001	<0.001	<0.001	16.125	6.349	103.981	<0.001	0.002	<0.001
Chl a + b	11.602	279.371	50.190	<0.001	<0.001	<0.001	3.935	94.816	17.781	0.018	<0.001	<0.001

#### 3.1.2 Effects on citrus leaf antioxidant enzymes

**Figure 4** presents the changes in citrus leaf antioxidant enzyme activities under different mulching and water-nitrogen treatments during 2023-2024. Analysis of variance showed that irrigation amount and nitrogen rate significantly affected the activities of all three enzymes in both 2023 and 2024 (*P* < 0.001), whereas mulching significantly affected only POD activity (*P* < 0.01). Among the interactions, W × N significantly affected only POD activity, while W × M and N × M significantly affected the activities of all three enzymes (*P* < 0.001) **Table 4**. Range analysis indicated that the order of factor importance for CAT and SOD was W > N > M, while for POD it was N > W > M. Both irrigation amount and nitrogen rate had pronounced dominant effects on the antioxidant enzymes, with mulching materials having a relatively minor effect. Overall, CAT, SOD, and POD exhibited similar response patterns. The activities of all three enzymes were generally highest at the low irrigation level and relatively lower at the moderate and high irrigation levels, indicating that water deficit was a key signal activating the antioxidant enzyme system. Moreover, enzyme activities were significantly higher under T7 (W3N1M3) than under T9 (W3N3M2), suggesting that sufficient nitrogen supply may be necessary to sustain high antioxidant enzyme activity. In both years, the activities of all three enzymes peaked under T7 and were significantly higher than those under the other treatments (*P* < 0.05), possibly owing to the combined effects of water-deficit-induced antioxidant defense and nutritional support from nitrogen. In 2023, CAT, SOD, and POD activities under T7 were 10.30%–55.21%, 6.36%–35.06%, and 13.15%–35.48% higher than the remaining treatments, respectively, with similar results in 2024. POD was most strongly affected by nitrogen rate (N > W > M), among treatments at the low irrigation level, high nitrogen levels maintained relatively high POD activity, while both the medium and low nitrogen levels led to a sharp decline, highlighting the central role of POD in the antioxidant defense system and its sensitivity to nitrogen availability.

**Figure 4:**
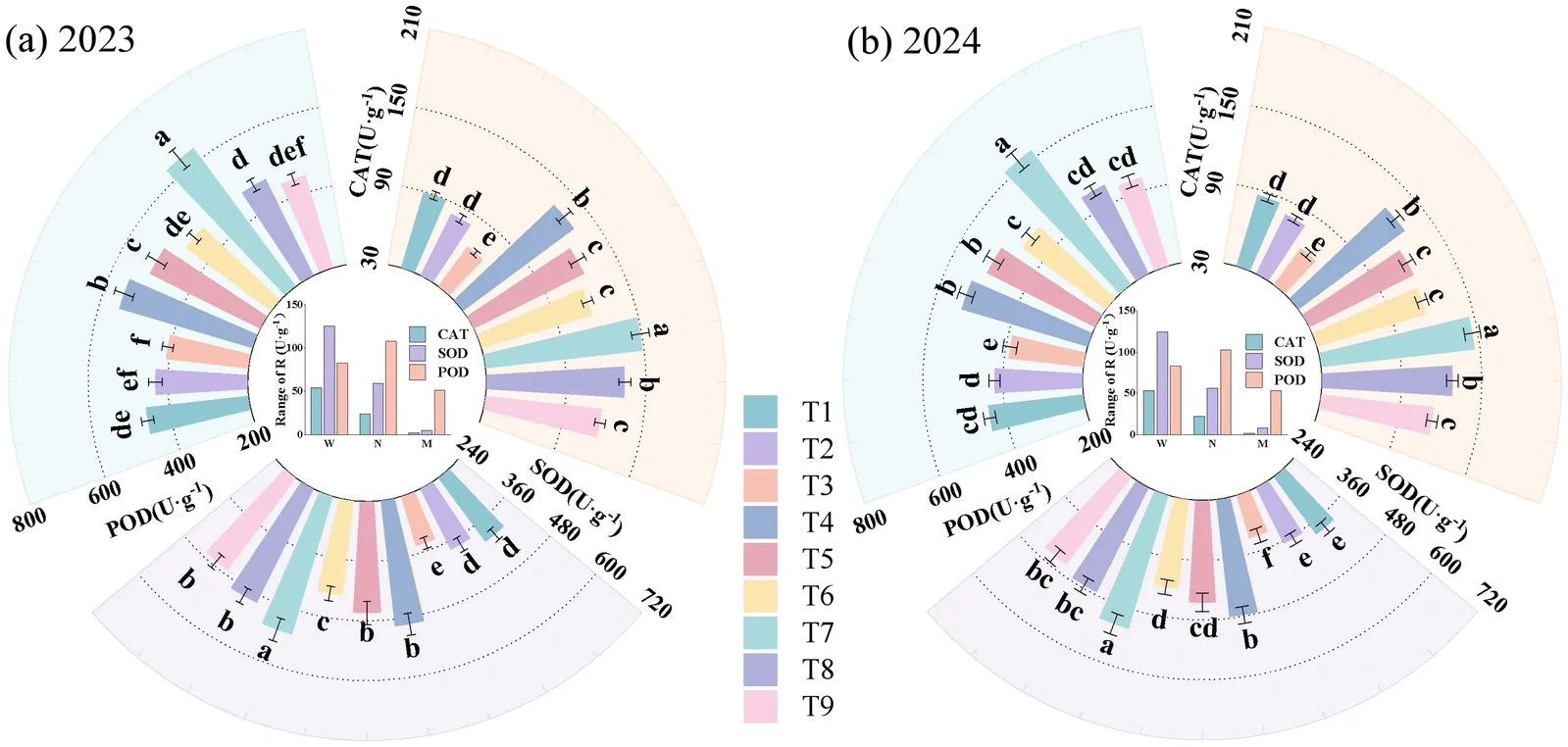
Effects of surface mulching and combined water-nitrogen application on citrus leaf antioxidant enzyme activities in 2023-2024. The inner bar chart represents the range (*R*) analysis values, and the outer bar chart represents the mean values of each treatment for different indicators. Different lowercase letters indicate significant differences among treatments (*P* < 0.05). Panels **(a, b)** represent cirus leaf antioxidant enzyme activities in 2023 and 2024, respectively.

**Table 4:** ANOVA results for antioxidant enzyme activities in citrus leaves.

Indicators	2023						2024					
	*F*-value			*P*-value			*F*-value			*P*-value		
	W	N	M	W	N	M	W	N	M	W	N	M
CAT	273.367	44.930	0.391	<0.001	<0.001	0.682	232.966	35.263	0.202	<0.001	<0.001	0.819
SOD	129.072	27.039	0.185	<0.001	<0.001	0.833	146.087	28.862	0.585	<0.001	<0.001	0.566
POD	24.461	35.779	8.752	<0.001	<0.001	0.002	23.818	28.973	8.453	<0.001	<0.001	0.002
	W×N	W×M	N×M	W×N	W×M	N×M	W×N	W×M	N×M	W×N	W×M	N×M
CAT	2.094	30.356	175.311	0.124	<0.001	<0.001	4.670	36.186	213.893	0.009	<0.001	<0.001
SOD	0.090	12.193	58.179	0.984	<0.001	<0.001	0.574	14.128	70.318	0.685	<0.001	<0.001
POD	13.896	40.410	29.306	<0.001	<0.001	<0.001	20.140	47.539	40.656	<0.001	<0.001	<0.001

#### 3.1.3 Effects on citrus photosynthetic parameters

**Figure 5** shows the changes in citrus leaf photosynthetic parameters under different mulching and water-nitrogen treatments during 2023-2024. Analysis of variance showed that irrigation amount significantly affected all six photosynthetic indices in both 2023 and 2024 (*P* < 0.001). Nitrogen rate significantly affected Pn, Tr, and Gs in both years and CE only in 2023, whereas the main effect of mulching material was nonsignificant for all indices. Regarding the interactions, W × N had no significant effects; W × M significantly affected Pn, Tr, Gs, and CE in 2023, but only Tr and Gs in 2024; and N × M had highly significant effects on most of the six indices (*P* < 0.001) **Table 5**. Range analysis indicated that irrigation amount was the primary factor affecting photosynthetic gas exchange parameters, exerting a greater influence on Pn, Tr, and Gs than nitrogen rate and mulching material (W > N > M). The differences among irrigation levels were more pronounced than those among nitrogen levels. In terms of the optimal treatment combination for each parameter, the high-irrigation and high-nitrogen treatment exhibited the best overall photosynthetic performance, with most photosynthetic parameters under silver-black double-color film (M3) being superior to those under the other two mulching types. Pn, Tr, and Gs decreased as irrigation and nitrogen levels decreased. All three parameters peaked in T1 (W1N1M1) and were significantly higher than under all other treatments (*P* < 0.05). In 2023, compared with T1, the remaining treatments decreased by 1.02%–24.36%, 3.18%–19.47%, and 11.02%–41.15%, respectively. In 2024, the corresponding decreases were 1.09%–17.41%, 4.64%–17.48%, and 8.22%–44.56%, indicating that high irrigation amount and high nitrogen rate promoted stronger carbon fixation capacity and photosynthesis. Ci decreased synchronously with Pn and other parameters under high and medium irrigation levels, but under low irrigation levels, Ci unexpectedly increased, being higher than the other two irrigation levels and contrasting with the decreasing trends of Pn and Gs. LWUE showed little overall variation; T4 (W2N1M2) had the highest value, while T1 remained at a relatively high level. Carboxylation efficiency (CE) generally decreased with reductions in irrigation amount and nitrogen rate, with CE under low irrigation level being notably lower than under the other two levels.

**Figure 5:**
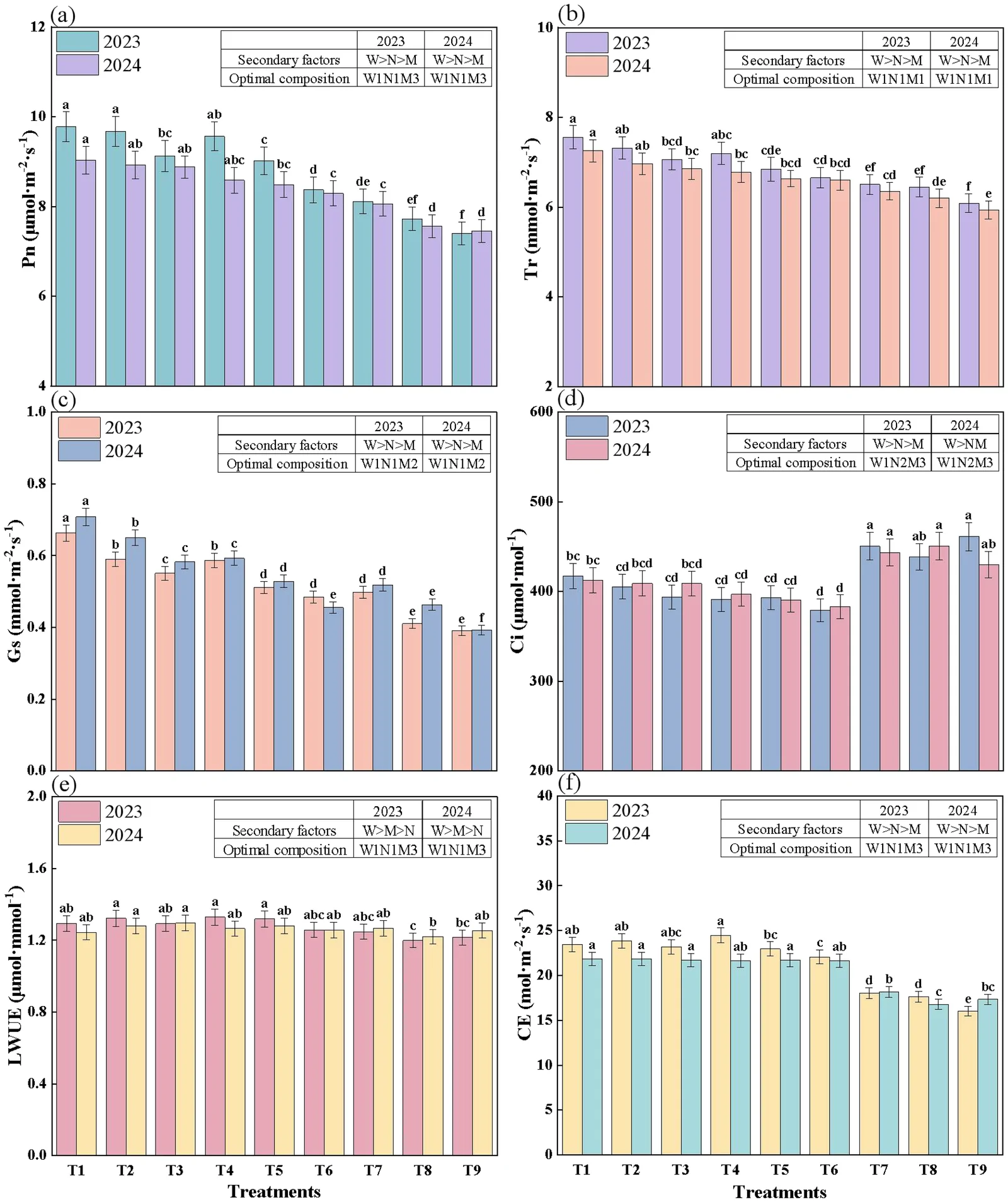
Effects of surface mulching and combined water-nitrogen application on citrus photosynthetic parameters in 2023-2024. The inserted tables show the range ranking and optimal combinations. Different lowercase letters indicate significant differences among treatments (*P* < 0.05). Panels **(a–f)** represent the mean values of Pn, Tr, Gs, Ci, LWUE, and CE in 2023–2024, respectively.

**Table 5:** ANOVA results for leaf photosynthetic parameters.

Indicators	2023						2024					
	*F*-value			*P*-value			*F*-value			*P*-value		
	W	N	M	W	N	M	W	N	M	W	N	M
Pn	91.023	20.131	1.782	<0.001	<0.001	0.194	47.512	3.775	1.154	<0.001	0.041	0.335
Tr	41.820	10.496	0.326	<0.001	<0.001	0.726	27.016	6.833	1.143	<0.001	0.005	0.339
Gs	210.581	91.276	0.065	<0.001	<0.001	0.937	255.916	117.979	0.064	<0.001	<0.001	0.938
Ci	38.409	0.746	0.661	<0.001	0.487	0.527	30.607	1.527	0.145	<0.001	0.241	0.866
LWUE	11.780	1.694	2.504	<0.001	0.209	0.107	3.524	0.413	2.827	0.049	0.667	0.083
CE	165.258	8.241	0.644	<0.001	0.002	0.536	113.623	0.992	0.913	<0.001	0.388	0.418
	W×N	W×M	N×M	W×N	W×M	N×M	W×N	W×M	N×M	W×N	W×M	N×M
Pn	0.933	9.395	42.086	0.467	<0.001	<0.001	0.707	1.931	22.348	0.598	0.149	<0.001
Tr	0.308	5.044	19.629	0.869	0.007	<0.001	0.547	3.125	12.267	0.703	0.041	<0.001
Gs	0.271	43.503	100.051	0.893	<0.001	<0.001	0.104	54.050	117.157	0.980	<0.001	<0.001
Ci	1.962	2.013	24.841	0.144	0.136	<0.001	0.472	1.150	15.403	0.756	0.365	<0.001
LWUE	1.134	0.768	5.313	0.372	0.560	0.005	1.347	0.246	1.665	0.291	0.908	0.202
CE	2.252	7.072	106.679	0.104	0.001	<0.001	1.058	1.099	58.469	0.405	0.387	<0.001

### 3.2 Effects of surface plastic film mulching and combined water and nitrogen application on citrus growth characteristics

#### 3.2.1 Effects on citrus new shoot growth

**Figure 6** illustrates the dynamic changes in citrus length of New shoot under different mulching and water-nitrogen treatments during 2023-2024. Analysis of variance showed that irrigation amount and nitrogen rate significantly affected new shoot growth in both years (*P* < 0.001), whereas the main effect of mulching material was not significant (*P* > 0.05). The W × M and N × M interactions were significant in both years (*P* < 0.001), while the W × N interaction was not significant (*P* > 0.05) **Table 6**. Range analysis showed that the range values for irrigation amount and nitrogen rate were much larger than that for mulching material, although their ranking varied between years (2023: W > N > M; 2024: N > W > M), with mulching having the smallest range in both years. Among the three mulching types, silver-black double-color film (M3) had the highest K-value, but the overall range for mulching was far smaller than those of the water and nitrogen factors, resulting in limited differences among mulching treatments. The New shoot growth trends were broadly similar across treatments; growth rates gradually accelerated as shoots developed, peaked in August during the topdressing period, and subsequently declined. T1 (W1N1M1) exhibited the highest maximum growth rate (9.98 mm·d^-1^), followed by T4 (W2N1M2), while T9 (W3N3M2) had the lowest (8.2 mm·d^-1^). New shoot length generally decreased with reductions in irrigation amount and nitrogen rate, with T1 (W1N1M1) being the most favorable treatment for shoot growth. In 2024, final shoot length was significantly greater in T7 (W3N1M3) than in T6 (W2N3M1) (*P* < 0.05), although its early-stage growth was slower. This confirms the critical roles of water and nitrogen in shoot growth: water promotes shoot initiation, while nitrogen promotes rapid shoot elongation.

**Figure 6:**
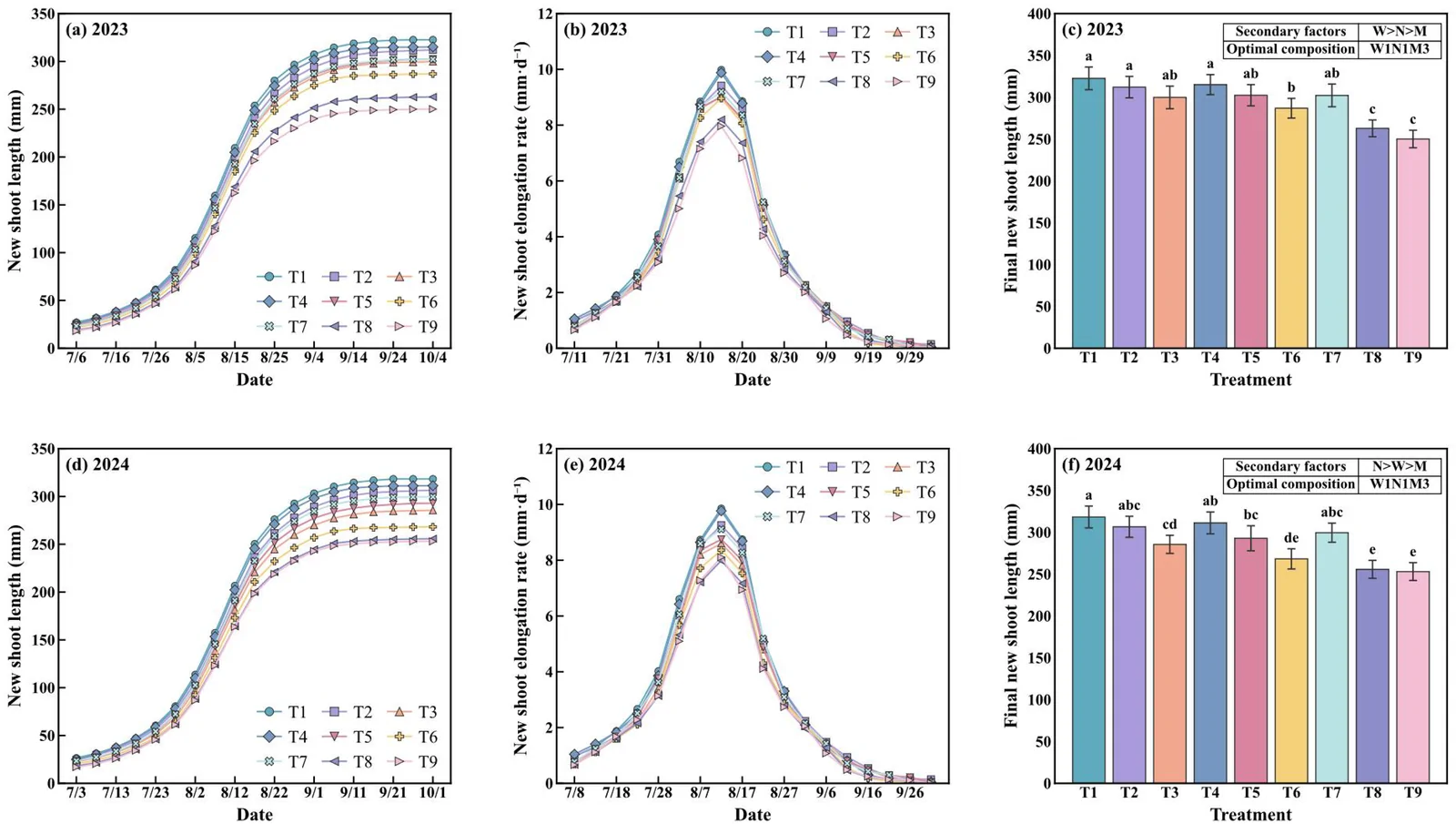
Effects of surface mulching and combined water-nitrogen application on citrus new shoots in 2023-2024. Panels **(a, d)** show the dynamic changes in new shoot length; panels **(b, e)** show the dynamic changes in new shoot growth rates; Panels **(c, f)** show the mean final shoot length for each treatment. Different lowercase letters indicate significant differences among treatments (*P* < 0.05).

**Table 6:** ANOVA results for new shoot growth.

Indicators	2023						2024					
	*F*-value			*P*-value			*F*-value			*P*-value		
	W	N	M	W	N	M	W	N	M	W	N	M
New shoot	24.488	17.117	1.905	<0.001	<0.001	0.175	18.207	25.905	2.457	<0.001	<0.001	0.111
	W×N	W×M	N×M	W×N	W×M	N×M	W×N	W×M	N×M	W×N	W×M	N×M
	1.664	9.539	13.355	0.202	<0.001	<0.001	1.600	13.083	9.313	0.217	<0.001	<0.001

#### 3.2.2 Effects on citrus fruit diameter at different growth stages

**Figure 7** presents the staged changes in citrus fruit diameter under different mulching and water-nitrogen treatments during 2023-2024. Analysis of variance showed that irrigation amount, nitrogen rate, and mulching material significantly affected both transverse and longitudinal fruit diameters in both years (*P* < 0.01 or *P* < 0.001) **Table 7**. All two-way interactions among the three factors also had significant effects on both diameter measurements (*P* < 0.01 or *P* < 0.001). Range analysis indicated that irrigation amount was the primary factor affecting final fruit diameter, with the maximum mean fruit diameter occurring under moderate deficit. The range values for nitrogen rate and mulching material were much smaller than that for irrigation amount, with silver-black double-color film (M3) having a higher K-value than white transparent film (M1) and black opaque film (M2). The effects of irrigation amount, nitrogen rate, and mulching material on fruit diameter were concentrated in the fruit enlargement stage (Stage III), which accounted for 59.84%-68.92% (longitudinal diameter) and 57.97%-67.27% (transverse diameter) of the total increments across the whole growth period in both years. Differences among treatments at the budding and flowering stage (Stage I) and the fruit coloration and sugar-accumulating stage (Stage IV) were relatively small. T5 (W2N2M3) had the largest final fruit diameter, significantly higher than under all other treatments (*P* < 0.05), with longitudinal diameters of 86.52 mm and 87.39 mm in 2023 and 2024, respectively, and transverse diameters of 82.10 mm and 79.88 mm. Compared with T5, the longitudinal and transverse diameters of the remaining treatments decreased by 4.62%–20.71% and 4.03%–26.31%, respectively. However, the fruit shape index of T5 was relatively low, while T9 (W3N3M2), despite having smaller fruit diameters, exhibited the best fruit shape index among all treatments (**Figures 7c, f**), indicating that the absolute size of fruit diameter does not necessarily correspond to a desirable fruit shape. Under high and medium irrigation levels, fruit diameter initially increased and then decreased with reductions in nitrogen rate, with the most pronounced fruit enlargement occurring at the medium nitrogen rate. Under the same nitrogen level, fruit diameter in the medium-irrigation treatment was generally larger than those in the high-irrigation and low-irrigation treatments, with the smallest fruit diameter observed under the combination of low irrigation amount and low nitrogen rate.

**Figure 7:**
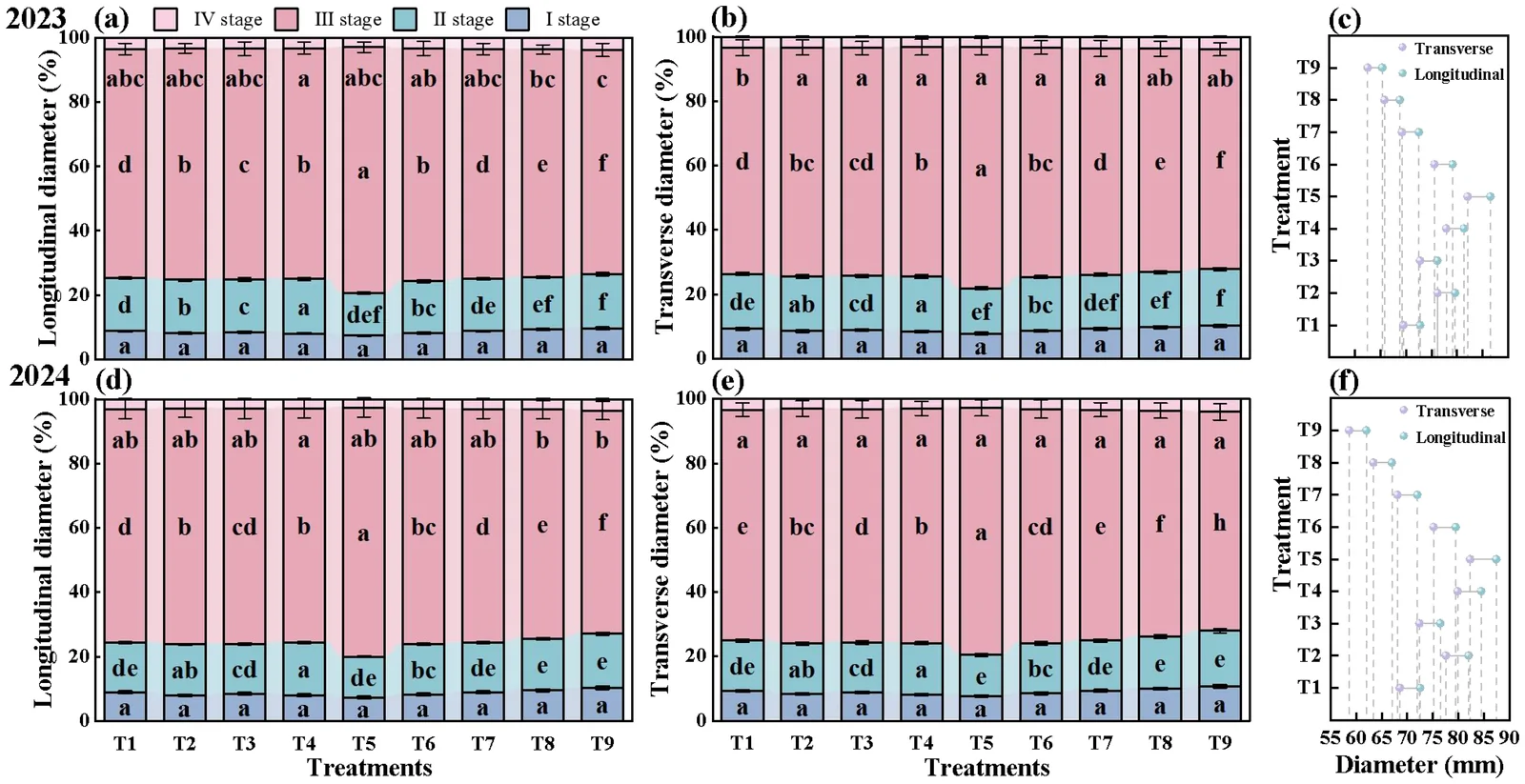
Effects of surface mulching and combined water–nitrogen application on citrus fruit diameter in 2023-2024. Panels **(a–d)** show the proportion of fruit diameter expansion at different growth stages; panels **(c, f)** show the final mean values of fruit diameter. Different lowercase letters indicate significant differences among treatments (*P* < 0.05).

**Table 7:** ANOVA results for fruit diameter.

Indicators	2023						2024					
	*F*-value			*P*-value			*F*-value			*P*-value		
	W	N	M	W	N	M	W	N	M	W	N	M
Transverse diameter	65.772	7.916	7.916	<0.001	0.003	0.003	66.061	8.646	7.215	<0.001	0.002	0.004
Longitudinal diameter	88.872	11.395	11.586	<0.001	<0.001	<0.001	55.386	7.382	6.180	<0.001	0.004	0.008
	W×N	W×M	N×M	W×N	W×M	N×M	W×N	W×M	N×M	W×N	W×M	N×M
Transverse diameter	5.858	5.858	39.305	0.003	0.003	<0.001	7.960	8.996	50.555	<0.001	<0.001	<0.001
Longitudinal diameter	8.651	8.535	55.737	<0.001	<0.001	<0.001	5.766	6.529	36.987	0.004	0.002	<0.001

### 3.3 Effects of surface plastic film mulching and combined water and nitrogen application on citrus yield and water-nitrogen use efficiency

**Figure 8** shows the effects of different mulching and water-nitrogen treatments on citrus yield and water-nitrogen use efficiency during 2023-2024. Analysis of variance showed that irrigation amount significantly affected single-fruit weight, fruit number, and yield in both years (*P* < 0.01 or *P* < 0.001). Nitrogen rate significantly affected these variables except yield in 2023, whereas mulching material significantly affected yield only. The W × M and N × M interactions significantly affected single-fruit weight, fruit number, and yield, while the W × N interaction significantly affected yield only in 2023 **Table 8**. Range analysis indicated that irrigation amount had the greatest impact on single fruit weight, fruit number, and yield (W > N > M), while mulching materials had the least impact. The optimal combinations for these three indicators were W2N1M3, W1N1M3, and W1N1M3, respectively. The response patterns of single fruit weight and fruit number diverged under different water and nitrogen conditions. T5 (W2N2M3) had the maximum single fruit weight, with values of 324.15 and 333.88 g in 2023 and 2024, respectively. In 2023, the low irrigation amount treatments (T7–T9) decreased by 6.88%–19.56% compared with T5, with similar reductions in 2024. Under high and medium irrigation levels, single fruit weight initially increased and then decreased with reductions in nitrogen rate, peaking at the medium nitrogen rate; under low irrigation level, single fruit weight continuously decreased as nitrogen rate decreased. Fruit number was highest in T1 (W1N1M1). In 2023, the remaining treatments decreased by 2.9%–23.3% compared with T1, and in 2024 by 2.0%–15.0%. Yield was highest under T1 (W1N1M1) and decreased with reductions in irrigation amount and nitrogen rate. The difference between the high-irrigation and medium-irrigation treatments was relatively small, but yield under the low-irrigation treatments decreased notably(*P* < 0.05). In 2023, the low-irrigation treatments (T7–T9) decreased by 14.85%–33.35% compared with T1, and in 2024 by 14.05%–29.88%.

**Figure 8:**
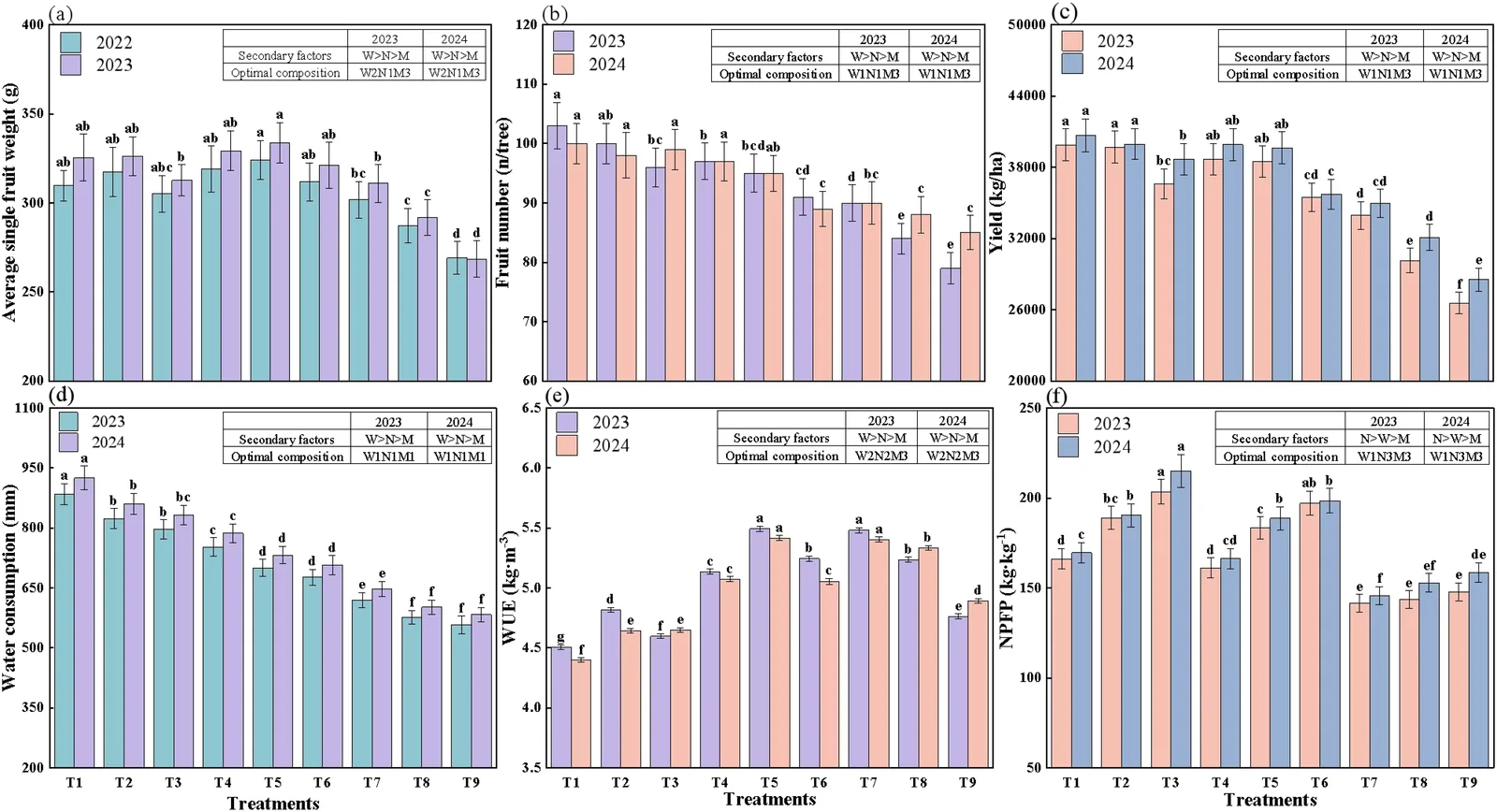
Effects of surface mulching and combined water-nitrogen application on citrus yield and water-nitrogen use efficiency in 2023-2024. The inserted tables show the range ranking and optimal combinations. Different lowercase letters indicate significant differences among treatments (*P* < 0.05). Panels **(a–f)** represent the mean values of single fruit weight, ftuit number, yield, water consumption, WUE, and NPFP in 2023–2024, respectively.

**Table 8:** ANOVA results for yield, water-use efficiency, and nitrogen partial factor productivity.

Indicators	2023						2024					
	*F*-value			*P*-value			*F*-value			*P*-value		
	W	N	M	W	N	M	W	N	M	W	N	M
Single fruit weight	21.724	5.383	1.621	<0.001	0.013	0.223	28.351	8.594	2.192	<0.001	0.002	0.138
Fruit number	57.146	15.125	0.664	<0.001	<0.001	0.526	27.966	4.768	1.192	<0.001	0.020	0.324
Yield	12.466	3.036	7.947	<0.001	0.071	0.003	105.495	27.336	5.131	<0.001	<0.001	0.016
Water consumption	326.050	31.126	0.298	<0.001	<0.001	0.746	226.050	31.126	0.298	<0.001	<0.001	0.664
WUE	25.339	6.576	7.815	<0.001	0.006	0.003	241.578	32.818	41.344	<0.001	<0.001	<0.001
NPFP	13.148	21.367	6.450	<0.001	<0.001	0.007	91.511	47.719	7.872	<0.001	<0.001	0.003
	W×N	W×M	N×M	W×N	W×M	N×M	W×N	W×M	N×M	W×N	W×M	N×M
Single fruit weight	1.853	3.910	12.844	0.163	0.019	<0.001	2.371	5.980	17.115	0.091	0.003	<0.001
Fruit number	0.583	7.475	27.504	0.679	<0.001	<0.001	1.218	3.045	14.897	0.338	0.044	<0.001
Yield	59.291	41.836	75.352	<0.001	<0.001	<0.001	2.843	13.620	51.553	0.055	<0.001	<0.001
Water consumption	0.323	14.698	152.374	0.874	<0.001	<0.001	0.307	14.698	152.374	0.869	<0.001	<0.001
WUE	323.219	301.484	642.809	<0.001	<0.001	<0.001	313.322	260.546	155.786	<0.001	<0.001	<0.001
NPFP	54.737	108.456	78.860	<0.001	<0.001	<0.001	6.052	29.578	55.434	0.003	<0.001	<0.001

Analysis of variance showed that irrigation amount and nitrogen rate significantly affected ET, WUE, and NPFP in both years (*P* < 0.001), whereas mulching material significantly affected only WUE and NPFP. The W × M and N × M interactions significantly affected all three variables, while W × N primarily affected WUE and NPFP. These results indicate that irrigation amount had the broadest influence, whereas mulching material regulated yield, WUE, and NPFP mainly through its interactions with irrigation amount and nitrogen rate **Table 8**. Range analysis indicated that irrigation amount had the greatest effect on ET and WUE (W > N > M), while nitrogen rate had the greatest effect on NPFP (N > W > M). The optimal combinations were W1N1M1, W2N2M3, and W1N3M3, respectively. Evapotranspiration (ET) decreased with reductions in irrigation amount and nitrogen rate. In 2023, the ET of the remaining treatments decreased by 6.95%–36.95% compared with T1, with similar reductions in 2024. WUE was highest under T5 (W2N2M3), reaching with values of 5.50 and 5.48 kg m^-3^ in 2023 and 2024, respectively, followed by T7 (W3N1M3), with corresponding values of 5.42 and 5.39 kg m^-3^. In 2023, compared with T5 and T7, the remaining treatments decreased by 4.57%–18.00% and 4.34%–17.80%, respectively, with similar reductions in 2024. Overall, the high-irrigation treatment had the lowest WUE; the medium-irrigation and low-irrigation treatments had notably higher WUE than the high-irrigation treatment(*P* < 0.05). Under the medium irrigation level, the medium nitrogen rate resulted in the highest WUE; under the low irrigation level, the high nitrogen rate resulted in the highest WUE. NPFP was highest for T3 (W1N3M3) and was significantly higher than that for all other treatments (*P* < 0.05), reaching 203.41 and 215.01 kg kg^-1^ in 2023 and 2024, respectively. Compared with T3, NPFP was 3.11%–30.44% lower in the other treatments in 2023 and 7.67%–32.68% lower in 2024.

### 3.4 Citrus growth-yield path analysis

Significant positive correlations were observed among the five citrus indicators-length of New shoot, fruit diameter, single fruit weight, fruit number, and yield (*P* < 0.001) (**Figure 9A**). Fruit number had the strongest correlation with yield (*r* = 0.94, *P* < 0.001), followed by single fruit weight (*r* = 0.92, *P* < 0.001). Length of New shoot was also positively correlated with fruit number (*r* = 0.88) and fruit diameter (*r* = 0.61). All indicators exhibited significant linear trends, satisfying the prerequisites for path analysis. Path analysis showed that length of New shoot had no direct effect on yield (*β* = 0.09, *P* > 0.05) but acted entirely through indirect pathways (total indirect effect = 0.86) (**Figure 9B**). Among the factors directly affecting yield, the total effect of fruit number (0.73) was greater than that of single fruit weight (0.46). The effect decomposition bar chart further quantified the direct, indirect, and total effects of each variable on yield (**Figure 9C**). The effects of length of New shoot and fruit diameter on yield were fully mediated, with zero direct effects. Fruit number and single fruit weight were the direct drivers of yield formation, with fruit number contributing more substantially.

**Figure 9:**
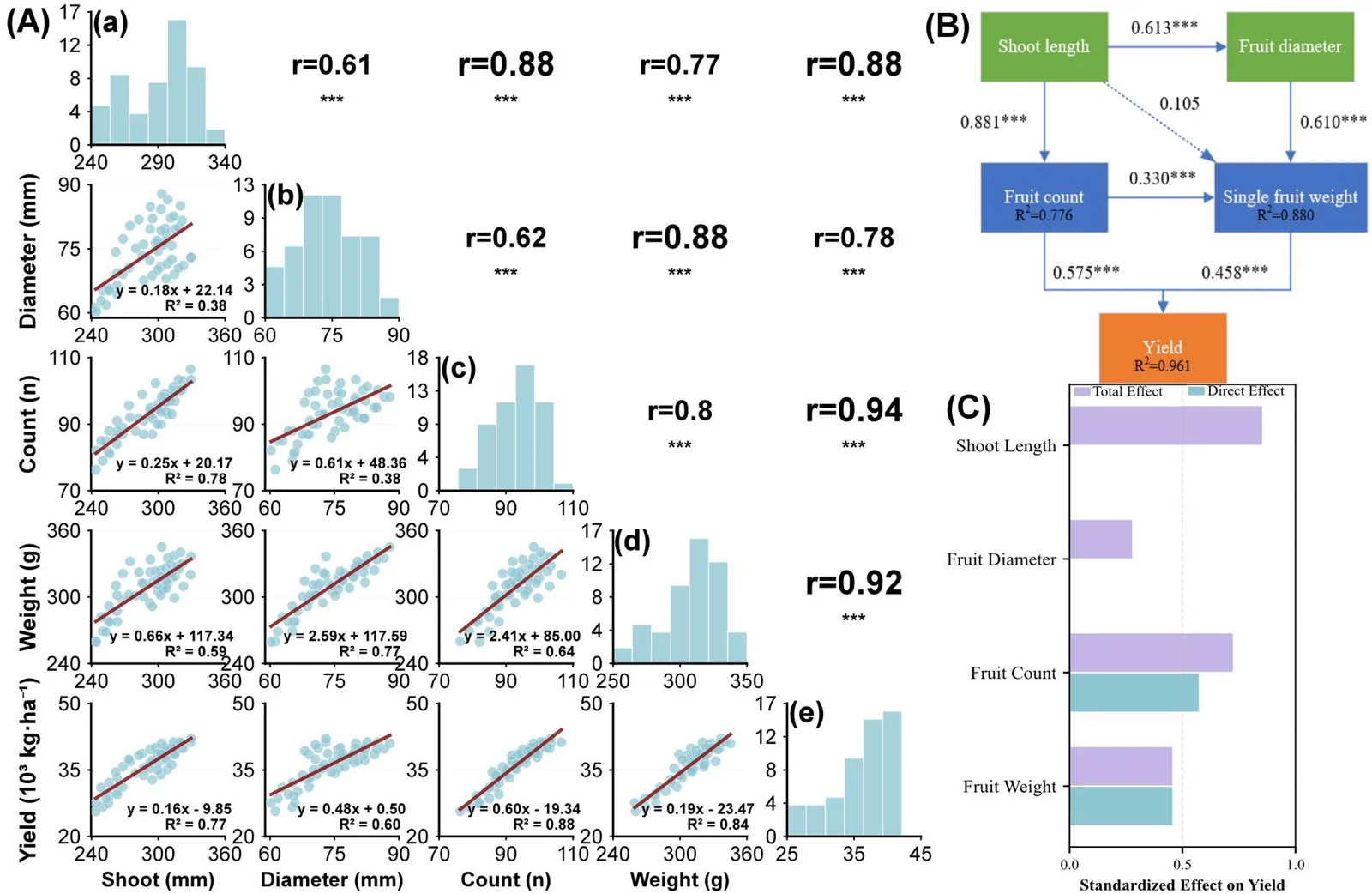
Path analysis of citrus growth characteristics and yield. Panel **(A)** shows data distribution and correlations among indicators. Panel **(B)** blue solid lines represent significant paths; blue dashed lines represent non-significant paths. Panel **(C)** shows the effect decomposition diagram. *, **, and *** indicate significance at the *P* < 0.05, *P* < 0.01, and *P* < 0.001 levels, respectively.

### 3.5 Response surface analysis and range optimization for yield, WUE, and NPFP under different mulching conditions

Based on a second-order polynomial regression model, the interactive effects of irrigation amount and nitrogen rate on yield, WUE, and NPFP under different mulching materials were analyzed. Water and nitrogen inputs significantly affected these three indicators under different mulching treatments, with coefficients of determination (*R²*) greater than 0.9 (**Table 9**). Under all three mulching conditions, yield increased continuously with increasing irrigation amount, while the effect of nitrogen rate on yield followed a parabolic curve, with yields approaching their maximum values near N = 200–220 kg·ha^-1^ for M1, M2, and M3. Under M3, yield peaked at 51,436 kg·ha^-1^ at W = 800 mm and N = 240 kg·ha^-1^ (**Figures 10**, **11a, d, g**). Under M1 and M2, WUE decreased with increasing irrigation amount, reaching its maximum at W = 450 mm, which aligns with the principle that moderate water deficit improves WUE. Under M3, the response of WUE to irrigation amount contrasted with that under M1 and M2. The effect of nitrogen rate on WUE was relatively moderate, with the optimum occurring near medium nitrogen rates (190–210 kg·ha^-1^) (**Figures 10**, **11b, e, h**). The response patterns of NPFP under the three mulching conditions were highly consistent (**Figures 10**, **11c, f, i**): NPFP continuously decreased with increasing nitrogen rate and continuously increased with increasing irrigation amount, reflecting the positive effect of water in promoting fertilizer efficiency. The maximum NPFP values occurred at the boundary of high irrigation and low nitrogen (W = 800 mm, N = 180 kg·ha^-1^). Using response surface spatial overlay analysis, thresholds of 95%, 90%, and 85% were applied to identify suitable multi-objective intervals for yield, WUE, and NPFP. No overlap was found at the 95% threshold, but overlapping regions appeared at the 85% and 90% thresholds. To balance high levels of all three indicators, 90% was selected as the comprehensive suitable interval threshold. Within the experimental domain, the model-estimated suitable water and nitrogen ranges for M1, M2, and M3 were: W = 687–800 mm, N = 180–208 kg·ha^-1^; W = 706–740 mm, N = 219–237 kg·ha^-1^; and W = 776–800 mm, N = 205–226 kg·ha^-1^, respectively (**Figure 12**).

**Table 9:** Multiple regression relationships between evaluation indicators and irrigation and fertilizer rates under different mulching materials.

Output variables	Regression equation	R^2^
Yield	Y_11_=-0.03732W^2^-1.222N^2^+0.3028WN+25.27W+293.7N-12472.5432	0.993 ***
	Y_12_=-0.03732W^2^-1.222N^2^+0.3028WN+19.67W+434.9N-38755.4621	
	Y_13_=-0.03732W^2^-1.222N^2^+0.3028WN+38.30W+450.7N-51238.0713	
WUE	Y_21_=-3.75856*10^-6^W^2^-3.28*10^-4^N^2^+1.9*10^-5^WN-5.82*10^-4^W+0.1175N-5.6812	0.920 ***
	Y_22_=-3.75856*10^-6^W^2^-3.28*10^-4^N^2^+1.9*10^-5^WN-1.684*10^-3^W+0.1367N-9.0835	
	Y_23_=-3.75856*10^-6^W^2^-3.28*10^-4^N^2^+1.9*10^-5^WN+1.731*10^-3^W+0.1430N-12.2153	
NPFP	Y_31_=-1.9*10^-4^W^2^-3.4*10^-3^N^2^+4.79*10^-4^WN+0.3486W+0.1582N+85.5902	0.994 ***
	Y_32_=-1.9*10^-4^W^2^-3.4*10^-3^N^2^+4.79*10^-4^WN+0.3155W+0.8454N-40.6067	
	Y_33_=-1.9*10^-4^W^2^-3.4*10^-3^N^2^+4.79*10^-4^WN+0.4024W+0.9101N-96.3786	
	Y_32_=-1.9*10^-4^W^2^-3.4*10^-3^N^2^+4.79*10^-4^WN+0.3155W+0.8454N-40.6067	
	Y_33_=-1.9*10^-4^W^2^-3.4*10^-3^N^2^+4.79*10^-4^WN+0.4024W+0.9101N-96.3786	

W, N, and M represent irrigation level, nitrogen application level, and mulching material, respectively. For all main effect tests, the numerator degrees of freedom were 2 and the error degrees of freedom were 20 (i.e., F(2,20)); for interaction tests, the numerator degrees of freedom were 4 and the error degrees of freedom were 18 (i.e., F(4,18)). ***indicates significance at the P<0.001 level.

**Figure 10:**
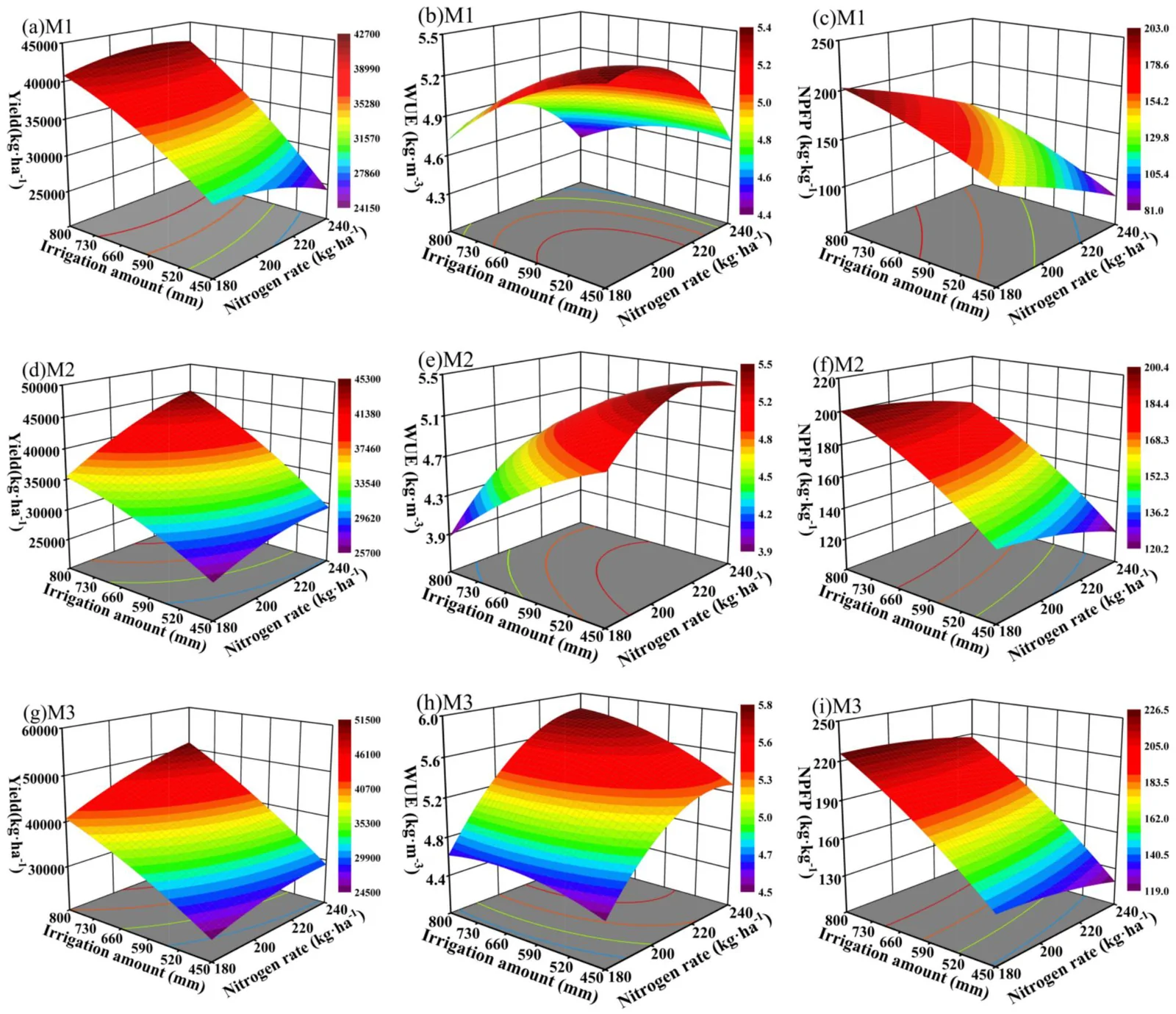
Response surface analysis of water-nitrogen-mulching coupling on yield and water-nitrogen use efficiency. The relationship of yield, WUE and NPFP with irrigation amount and nitrogen rate under M1 **(a, d, g)**, M2 **(b, e, h)** and M3 **(c, f, i)**.

**Figure 11:**
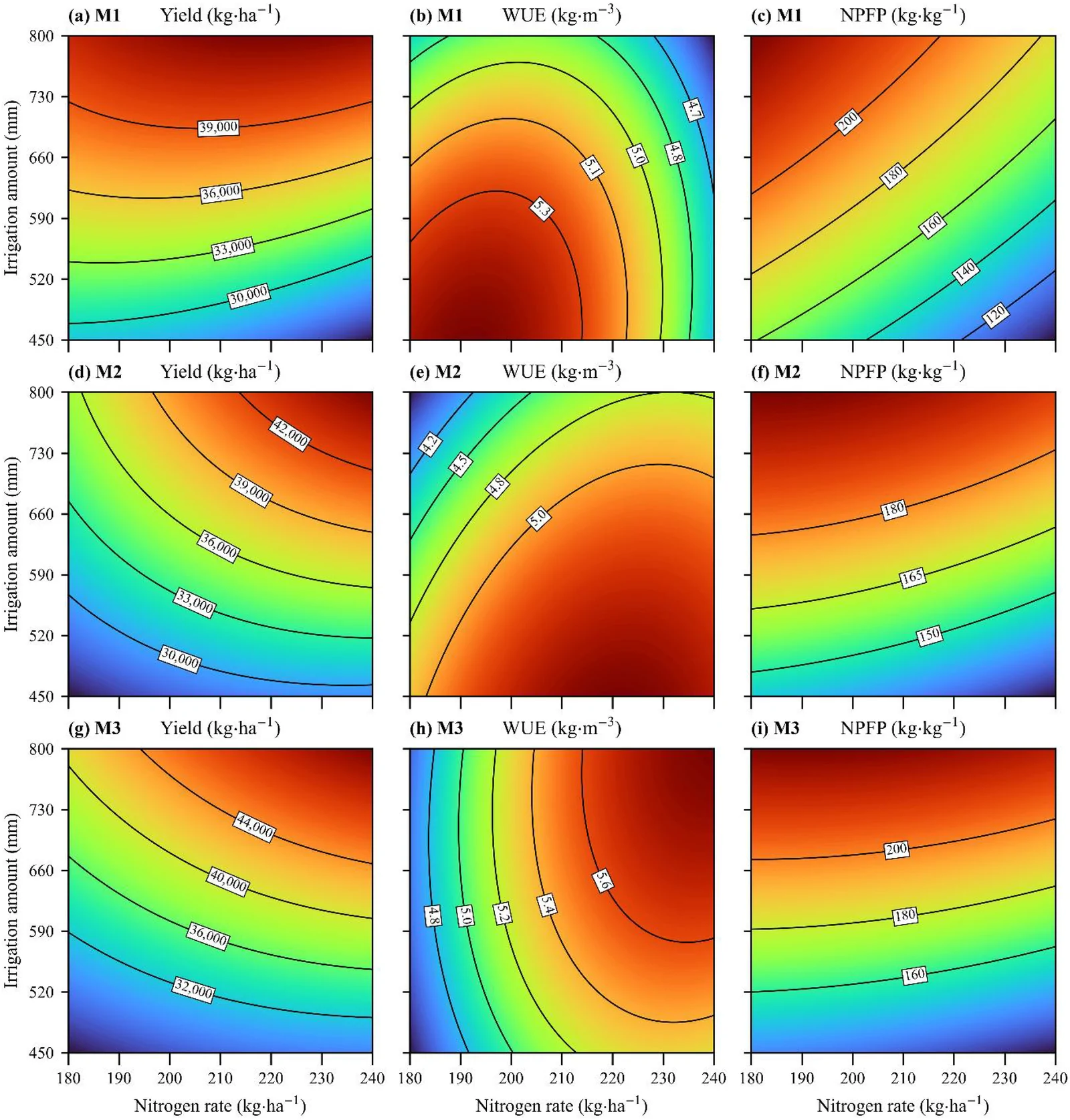
Contour plots of yield, WUE, and NPFP under different mulching materials.

**Figure 12:**
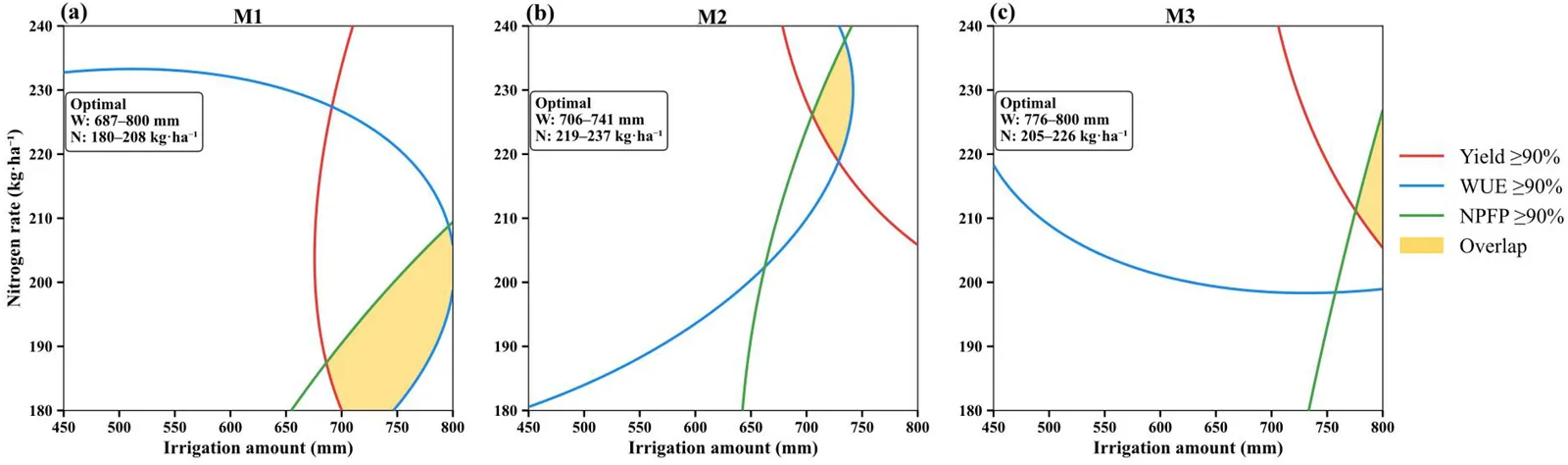
Comprehensive evaluation of suitable water-nitrogen intervals based on multi-objective spatial overlay. The overlapping intervals with the threshold of 90% of maximum yield, WUE and NPFP under M1 **(a)**, M2 **(b)** and M3 **(c)**.

### 3.6 Path analysis of water-nitrogen-mulching coupling effects

#### 3.6.1 Mantel test

**Figure 13** presents the Mantel test results between yield, WUE, NPFP, and various indicators in 2023-2024. Except for the three antioxidant enzymes (CAT, SOD, POD), yield was significantly correlated with all other indicators (*P* < 0.01). WUE was correlated only with the three antioxidant enzymes, while NPFP showed high correlations with most indicators. Furthermore, photosynthetic gas exchange parameters (Pn, Tr, Gs), fruit diameter (FD), chlorophyll components (Chl a, Chl b, Car, Chl a+b), and single fruit weight (SFW) mostly exhibited significant or highly significant positive correlations among themselves. This suggests that enhanced photosynthetic capacity effectively promotes leaf development and dry matter accumulation. Notably, antioxidant enzyme activities (CAT, SOD, POD) showed weak or even negative correlations with photosynthetic indicators (Pn, Gs) and SFW in most combinations, potentially reflecting energy or metabolic trade-offs between photosynthesis and antioxidant defense systems under stress conditions. Yield exhibited highly significant positive driving effects with leaf photosynthetic indicators (Pn, Tr, Gs), growth indicators (FSL, FD), chlorophyll content, and SFW (*P* < 0.01). This indicates that improving photosynthetic carbon assimilation capacity and leaf area, thereby promoting dry matter accumulation, is the core physiological pathway for achieving high yield. The significant pathways for WUE and NPFP were fewer than those for yield, but they were also evidently influenced by some photosynthetic traits, leaf traits, and SFW.

**Figure 13:**
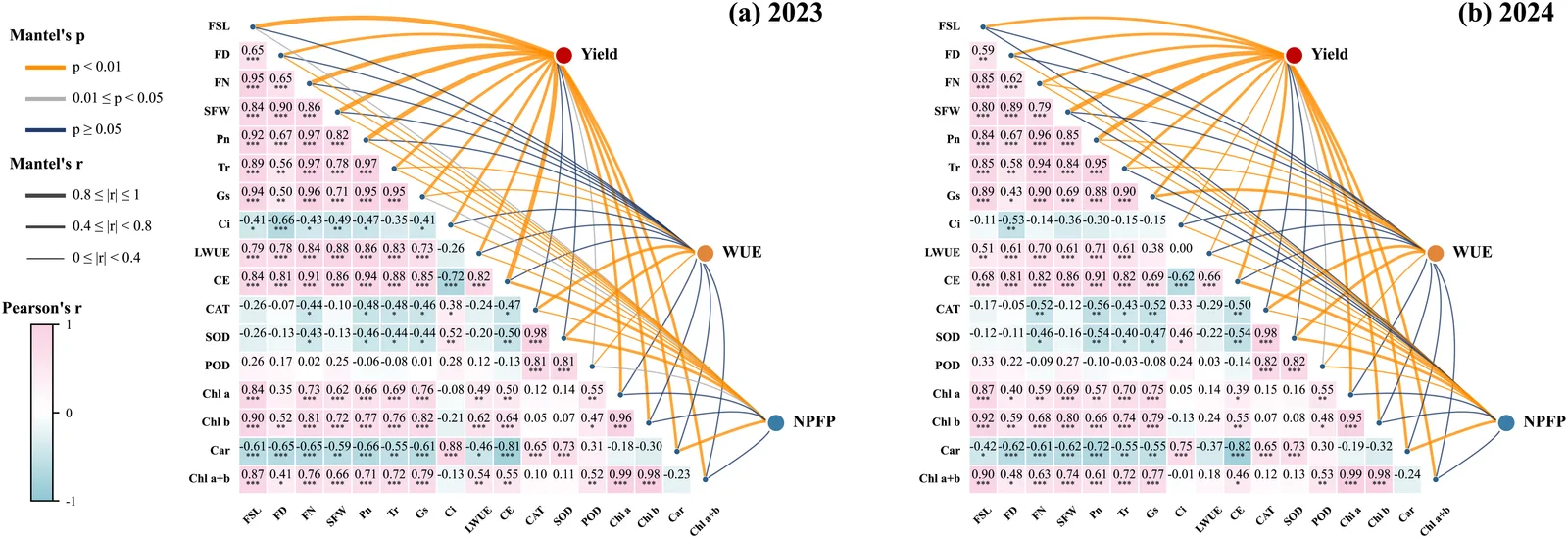
The Mantel test of each indicator in 2023 **(a)** and 2024 **(b)**. FSL, FD, FN, and SFW represent length of new shoot, fruit diameter, fruit number, and single fruit weight, respectively. *, **, and *** indicate significance at the *P* < 0.05, *P* < 0.01, and *P* < 0.001 levels, respectively. The relationship of yield, WUE and NPFP with irrigation amount and nitrogen rate under M1 **(a, d, g)**, M2 **(b, e, h)** and M3 **(c, f, i)**.

#### 3.6.2 Path analysis of water-nitrogen-mulching coupling effects

Partial Least Squares Path Modeling (PLS-PM) was used to analyze the pathways through which irrigation amount, nitrogen rate, and mulching material affect photosynthetic characteristics, growth, yield, WUE and NPFP (**Figure 14a**). Measurement model evaluation showed that the composite reliability (CR) values for the photosynthetic-trait (Pn and Gs) and growth (FN, FSL, and SFW) constructs were 0.953 and 0.956, respectively, while their average variance extracted (AVE) values were 0.911 and 0.878, respectively. These values met the requirements for construct reliability and convergent validity (**Table 10**). The heterotrait–monotrait ratio of correlations (HTMT) between the two constructs was 0.839, and the standardized root mean square residual (SRMR) was 0.067, indicating acceptable model fit. he outer collinearity assessment of the observed indicators is presented in **Table 11**. All indicator variance inflation factor (VIF) values were below 5, indicating no serious multicollinearity **Table 12**. The path coefficients and their significance are presented in **Figure 14b**. The 95% confidence intervals of all significant paths did not include zero, whereas those of the non-significant paths included zero, indicating that the estimated path coefficients were relatively stable.Path analysis revealed that irrigation amount had the strongest positive effect on photosynthetic indicators (*β* = 0.87, *P* < 0.01), followed by nitrogen rate (*β* = 0.33, *P* < 0.01). The direct effects of M3 and M2, with M1 as the reference level, were not significant. Photosynthetic indicators had a highly significant direct effect on growth indicators (*β* = 0.93, *P* < 0.01), and growth indicators significantly positively affected yield (*β* = 0.78, *P* < 0.01). The direct effects of irrigation amount and nitrogen rate on yield were weaker than the direct effect of growth indicators on yield, indicating that water and nitrogen supply primarily drive yield formation by improving leaf photosynthetic function and promoting vegetative growth. The *R²* values for photosynthetic indicators, growth indicators, yield, WUE, and NPFP were 0.94, 0.87, 0.96, 0.87, and 0.99, respectively. The exceptionally high *R*² for yield indicates that photosynthetic and growth indicators nearly fully explained the yield variation in this experiment. The total effect of irrigation amount on yield was the highest (0.86), followed by photosynthetic indicators (0.73) and growth indicators (0.78). The total effects of M3 and M2 were 0.10 and 0.09, respectively, both implemented indirectly through the photosynthesis-growth pathway. The total effect of nitrogen rate was relatively low (0.15), and its direct effect on yield was not significant, mainly acting indirectly through photosynthesis (**Figure 14c**). The indirect effects of irrigation amount and nitrogen rate were greater than their direct effects, further confirming that photosynthetic characteristics and growth indicators are key mediating pathways through which water and nitrogen influence yield.

**Table 10:** Construct reliability and convergent validity.

Indicators	Cronbach's alpha	CR	AVE
Photosynthetic indicators	0.902	0.953	0.911
Growth indicators	0.930	0.956	0.878

**Table 11:** Heterotrait–monotrait ratio (HTMT) of correlations among the latent constructs.

Indicators	Photosynthetic indicators
Growth indicators	0.839

**Table 12:** Collinearity assessment of measurement indicators using variance inflation factors (VIFs).

Latent variable	Indicators	Outer VIF
Photosynthetic indicators	Pn	3.084
	Gs	3.084
Growth indicators	FSL	4.702
	FN	4.819
	SFW	2.918

**Figure 14:**
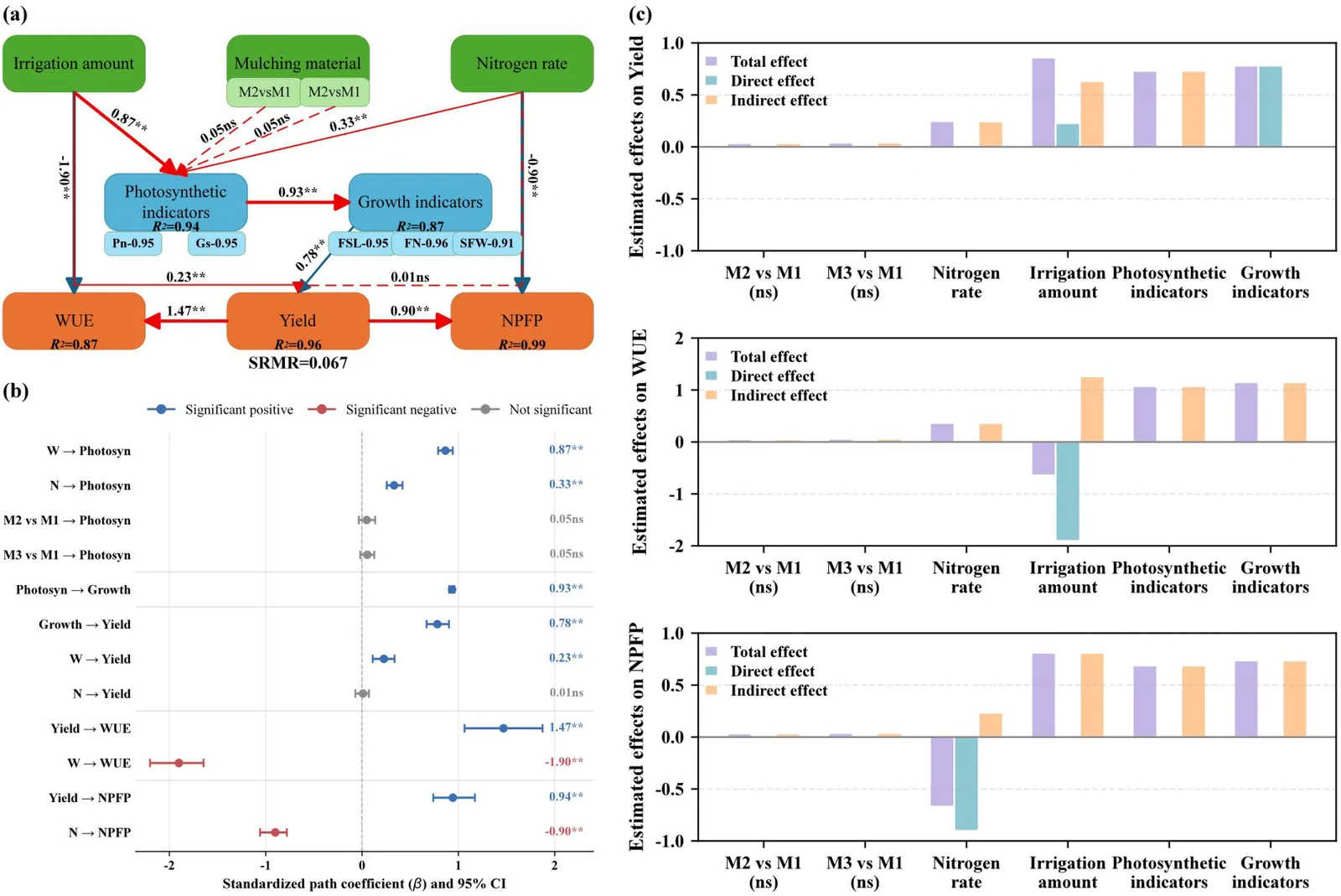
Path analysis of water-nitrogen-mulching coupling effects. Panel **(a)** shows the partial least-squares path model (PLS-PM). Solid red lines indicate significant positive paths, dashed red lines indicate nonsignificant positive paths, solid blue lines indicate significant negative paths, and dashed blue lines indicate nonsignificant negative paths. Panel **(b)** shows a forest plot of the standardized path coefficients and their 95% confidence intervals (CIs); points represent the standardized path coefficients, and horizontal lines represent the 95% CIs. Panel **(c)** shows the distribution of effects as a bar chart. * and ** indicate significance at *P* < 0.05 and *P* < 0.01, respectively; ns indicates nonsignificance (*P* > 0.05).

## 4 Discussion

### 4.1 Effects of surface plastic film mulching and combined water and nitrogen application on citrus physiological characteristics

In this study, the responses of citrus leaf photosynthetic parameters to water and nitrogen regulation exhibited a staged shift from “stomatal limitation” to “non-stomatal limitation.” Reduced irrigation triggered stomatal closure induced by root ABA signals, leading to synchronous declines in Gs, Tr, and Pn; reduced nitrogen application weakened carbon assimilation capacity by limiting chlorophyll synthesis and the renewal of photosynthetic enzyme proteins (Mu and Chen, 2021). The continuous decrease in CE with reductions in irrigation amount and nitrogen rate indicated dual constraints at the carboxylation site—specifically CO_2_ supply and Rubisco enzyme activity. During the mild-to-moderate deficit stages, the decline in Pn and CE was primarily due to insufficient CO_2_ supply caused by Gs reduction. This led to an over-reduction of the photosynthetic electron transport chain, triggering a burst of reactive oxygen species (ROS) and the oxidative degradation of chlorophyll (Wang et al., 2017; Qiao et al., 2024; Zhang et al., 2020). During this period, Ci decreased synchronously with Gs, with the dominant limiting factor for photosynthesis located at the “stomatal end.” As the deficit intensified further, Ci began to rise, contrasting sharply with the continuous decline in Pn and Gs. This rise in Ci marked a shift in photosynthetic limitation from stomatal factors to non-stomatal factors: photosynthetic enzyme activity was substantially impaired, and the assimilation capacity for CO_2_, rather than its diffusive supply, became the true bottleneck for photosynthesis (Gasque et al., 2016). The anomalous increase in carotenoids under severe deficit represents a photoprotective compensation mechanism activated when carbon assimilation is impeded and the electron transport chain is over-reduced—protecting the residual photosynthetic membrane system by enhancing thermal dissipation (Baccari et al., 2020).

Mulching played a critical regulatory role in these processes, with M3 generally performing best. The silver surface of the silver-black double-color film can reflect visible light upward, increasing photosynthetically active radiation in the lower canopy; the black surface blocks light penetration, effectively suppressing soil surface evaporation and moderating soil temperature fluctuations (Zhao et al., 2023). In this experiment, the reductions in Gs, Pn, and CE under water and nitrogen stress were smallest in the treatments with M3, indicating that the silver-black film alleviated photosynthetic inhibition induced by deficit irrigation and nitrogen reduction by maintaining higher stomatal conductance and photosynthetic enzyme activity (Yang et al., 2023). The differences in photosynthetic responses among the three mulching materials under different water-nitrogen combinations reflect the coupling regulatory effects between mulching and water-nitrogen supply.

### 4.2 Effects of surface plastic film mulching and combined water and nitrogen application on citrus growth characteristics

This study found that citrus New shoot growth and fruit diameter development were synergistically regulated by irrigation amount, nitrogen rate, and mulching material, exhibiting a close “source-sink” trade-off relationship during growth and resource allocation. Water deficit constrains New shoot elongation at both physical and metabolic levels. At the physical level, insufficient cell turgor directly limits the longitudinal expansion of newly formed cells in the apical meristem. At the metabolic level, water deficit reduces the mass flow and diffusion flux of soil nitrogen to the root surface, limiting nitrogen-dependent protein synthesis and cytokinin synthesis in meristematic tissues and leading to shortened internodes (Plett et al., 2020; Kudoyarova et al., 2015). Reduced nitrogen application further exacerbates this limitation by restricting cell division activity in the apical meristem (Luo et al., 2020). The high-irrigation and high-nitrogen treatment (T1, W1N1M1), benefiting from high water-nitrogen inputs and micro-environmental improvements from white transparent film mulch, exhibited the strongest carbon assimilation capacity, preferentially translocating photosynthetic products to the apical meristem and achieving the highest shoot growth rate. Conversely, the low-irrigation and low-nitrogen treatment (T9, W3N3M2), subjected to dual water and nitrogen deficits, shifted its carbon metabolism from “promoting growth” to “ensuring survival,” allocating assimilates primarily for osmotic adjustment and stress defense, resulting in significant shoot growth inhibition.

The fruit enlargement stage is a critical period for pulp cell division and juice sac expansion, during which the fruit is extremely sensitive to changes in water and nitrogen (Chen et al., 2022). Moderate water restriction can inhibit excessive vegetative growth, reduce assimilate competition between New shoots and fruits, and combined with appropriate reductions in nitrogen supply, which can increase the proportion of photosynthetic products allocated to fruits, thereby promoting fruit diameter enlargement (Yang et al., 2022). However, when nitrogen application continues to decline to severely deficient levels, chlorophyll synthesis and Rubisco enzyme protein renewal are hindered, leading to a substantial reduction in total photosynthetic carbon assimilation. Fruit development becomes restricted due to insufficient carbon skeleton supply, causing fruit diameter to shrink (Tantray et al., 2020). When water and nitrogen supplies are severely inadequate, cell turgor insufficiency directly inhibits the physical expansion of juice sac cells, while carbon assimilate supply is markedly reduced, resulting in a continuous decrease in fruit diameter with declining nitrogen rates (Hernandez-Santana et al., 2021). Mulching materials played a key buffering and stabilizing regulatory role in both processes by modulating soil hydrothermal conditions and the root zone micro-environment. These findings suggest that the selection of appropriate mulching materials combined with precise water-nitrogen synergistic management is a key technical pathway for preventing excessive vegetative growth or restricted fruit development.

### 4.3 Effects of surface plastic film mulching and combined water and nitrogen application on citrus yield and water-nitrogen use efficiency

Yield is synergistically determined by fruit number and single fruit weight, which exhibit pronounced trade-off differentiation under different water-nitrogen ratios. Under mild deficit, fruit load was high but single fruit weight was limited. As deficit intensified to moderate levels, the tree initiated physiological fruit drop to reduce sink organ number, concentrating assimilates on retained fruits and thereby increasing single fruit weight (Primo-Capella et al., 2024; Chen et al., 2025). This reflects the source-sink regulation strategy under continuous stress. Meanwhile, reduced nitrogen application weakened carbon assimilation capacity (Huang et al., 2022), exacerbating product competition among multiple sinks and amplifying the trade-off between fruit number and single fruit weight. In this study, T1 (W1N1M1) achieved the highest yield through the highest fruit number and a relatively high single fruit weight. Conversely, T9 (W3N3M2), suffering from dual water and nitrogen deficits, experienced significant concurrent declines in fruit number and single fruit weight, resulting in the lowest yield. The optimal WUE in T5 (W2N2M3) was attributed to the silver-black double-color film reducing non-productive transpiration and enhancing photosynthetic efficiency, combined with moderate deficit irrigation causing a greater reduction in ET than in yield, thereby maximizing WUE. The highest NPFP in T3 (W1N3M3) was attributed to the synergistic effects of high irrigation and the moisture-retaining properties of the silver-black double-color film. Sufficient irrigation maintained high soil water content and drove nitrogen mass flow and diffusion toward the root surface, while mulching slowed root zone water loss by suppressing surface evaporation. These effects collectively promoted root nitrogen uptake, ensured effective nitrogen supply under low nitrogen input, and achieved relatively high yield with minimal nitrogen application, thereby maximizing nitrogen output efficiency (Alkhateeb et al., 2024; Zhang et al., 2021).

### 4.4 Multi-objective optimization decisions for water-nitrogen-mulching coupling

Response surface analysis based on a second-order polynomial regression model revealed that under all three mulching materials, yield increased continuously with increasing irrigation amount without reaching a peak within the experimental range, indicating that the irrigation effect had not yet saturated. WUE and yield exhibited an inherent optimization conflict: the maximum WUE occurred in the low irrigation zone, but the yield in this area was far lower than that in the high irrigation zone, indicating that solely pursuing high WUE may severely sacrifice yield. NPFP decreased with increasing nitrogen application across all treatments, with its maximum in the high irrigation and low nitrogen zone, whereas the optimal yield zone favored high irrigation and high nitrogen, reflecting the opposite nitrogen demands of these two objectives. The key to improving NPFP lies not in simply reducing nitrogen input, but in maximizing yield within a reasonable nitrogen application range to compensate for the nitrogen input through yield gains. Under the three mulching conditions, yield, WUE, and NPFP exhibited acceptable compatible intervals after appropriately relaxing the threshold to 90%. Within the experimental domain, the suitable water and nitrogen ranges for M1, M2, and M3 were as follows: W = 687–800 mm, N = 180–208 kg·ha^-1^; W = 706–740 mm, N = 219–237 kg·ha^-1^; and W = 776–800 mm, N = 205–226 kg·ha^-1^, respectively. Considering the optimization directions of the three indicators, M3 excels in stronger photosynthetic driving force, better growth performance, and higher yield potential, but realizing its high-yield potential requires higher water and nitrogen inputs. M1 offers a broader suitable water-nitrogen range but has the lowest yield among the three, making it more operationally feasible under the overall strategy of deficit irrigation and nitrogen reduction. M2 has the narrowest suitable water–nitrogen range, requiring the most stringent water-nitrogen coordination. Therefore, M3 is preferred when water and nitrogen resources are sufficient to pursue maximum yield; M1 is a lower-risk choice for balancing high yield and efficiency when resources are constrained; and M2 can be selected when management is precise and a balance between yield and efficiency is sought.

### 4.5 Path analysis of water-nitrogen-mulching coupling effects

This study employed observed-variable path analysis and PLS-PM to elucidate the driving mechanisms of yield formation. Path analysis focused on the internal relationships among growth indicators, while PLS-PM integrated irrigation, nitrogen, and mulching into a unified framework to deconstruct the complete causal chain through which treatment factors drive yield via physiological mediators. Both methods pointed to the same core transmission pathway: photosynthesis drives growth, and growth determines yield. In path analysis, the total effect of length of New shoot on yield (0.86) was almost entirely realized through indirect pathways, with 59% via fruit number to yield and 20% via fruit diameter to single fruit weight to yield. This essentially reflects two physiological channels through which vegetative growth contributes to yield: supporting fruit set quantity and fruit enlargement. The more vigorous the New shoots, the larger the canopy photosynthetic area and the stronger the carbon assimilation capacity, thus providing more sufficient assimilates for fruit development (Li et al., 2024). In PLS-PM, irrigation amount promoted growth indicators by enhancing photosynthetic characteristics, thereby determining yield formation. The direct effect of photosynthetic characteristics on growth indicators was 0.94, and the direct effect of growth indicators on yield was 0.78. Although water and nitrogen can directly promote growth through hormone signal induction and cell turgor maintenance, under the conditions of this experiment, their growth-driving effects were primarily realized indirectly through the carbon skeletons provided by photosynthetic carbon assimilation. The two analytical chains are logically isomorphic; the vegetative growth state represented by New shoots in the path analysis corresponds highly to the core connotation of the latent variable “growth indicators” in PLS-PM. Both consistently showed that fruit number exerted a stronger driving effect on yield than single fruit weight. Furthermore, PLS-PM revealed that the direct negative effect of irrigation amount on WUE (-1.90) was stronger than its positive yield-increasing effect (1.47). It contrasts with the conclusion by Wang et al (Wang et al., 2026). in arid-region potato studies that “as long as the yield increase is large enough, the positive effect can offset the water cost,” reflecting the differences in water-yield trade-offs across different crops and experimental conditions. The two methods, starting from different analytical levels, converged on the same physiological framework, providing cross-method validation for the reliability of the conclusions.

## 5 Conclusions

Irrigation amount and nitrogen rate were the dominant factors regulating photosynthetic pigment content and antioxidant enzyme activity in citrus leaves, while mulching had a relatively minor influence. T1 (W1N1M1) had the highest chlorophyll a and b contents, while carotenoids peaked in T9 (W3N3M2); T7 (W3N1M3) exhibited the highest activities of all antioxidant enzymes, which decreased sharply under the low-irrigation and low-nitrogen combination. Irrigation amount was the primary factor affecting photosynthetic parameters; T1 (W1N1M1) had the highest Pn, Tr, and Gs, while Ci peaked in T9 (W3N3M2). New shoot length and fruit diameter showed similar responses to water and nitrogen supply, suggesting that appropriate water and nitrogen inputs favored balanced shoot and fruit growth. Irrigation amount and nitrogen rate were the main factors influencing both, with the largest fruit diameter occurring under medium irrigation and medium nitrogen, and growth constrained under low irrigation and low nitrogen. Within the experimental domain, the suitable water and nitrogen ranges for M1, M2, and M3 were as follows: W = 687–800 mm, N = 180–208 kg·ha^-1^; W = 706–740 mm, N = 219–237 kg·ha^-1^; and W = 776–800 mm, N = 205–226 kg·ha^-1^, respectively. White transparent film (M1) had a broader suitable water–nitrogen range but the lowest yield among the three, offering stronger operational feasibility under the overall strategy of deficit irrigation and nitrogen reduction. Black opaque film (M2) performed lower than M1 and M3 for most indicators and had a narrow suitable water–nitrogen range, indicating relatively limited applicability. Silver-black double-color film (M3) excelled in stronger photosynthetic driving force and higher yield potential, but required higher irrigation and nitrogen inputs to achieve its potential. Path analysis and PLS-PM results demonstrated that photosynthesis and growth acted as mediators through which water, nitrogen, and mulching influenced yield; these three factors primarily affected photosynthesis, thereby indirectly regulating growth and subsequently determining yield formation. Fruit number contributed more to yield than single fruit weight.

## Data Availability

The original contributions presented in the study are included in the article/supplementary material. Further inquiries can be directed to the corresponding author.
